# Membrane protective role of autophagic machinery during infection of epithelial cells by *Candida albicans*

**DOI:** 10.1080/19490976.2021.2004798

**Published:** 2022-01-27

**Authors:** Pierre Lapaquette, Amandine Ducreux, Louise Basmaciyan, Tracy Paradis, Fabienne Bon, Amandine Bataille, Pascale Winckler, Bernhard Hube, Christophe d’Enfert, Audrey Esclatine, Elisabeth Dubus, Marie-Agnès Bringer, Etienne Morel, Frédéric Dalle

**Affiliations:** a Univ. Bourgogne Franche-Comté, Agrosup Dijon, UMR PAM A 02.102, Dijon, France; b Laboratoire de Parasitologie-Mycologie, Plateforme de Biologie Hospitalo-Universitaire Gérard Mack, Dijon, France; c CellImaP Corefacility, INSERM LNC-UMR1231, Dijon, France; d Dimacell Imaging Facility, Agrosup Dijon, INRA, INSERM, Univ. Bourgogne Franche-Comté, Dijon, France; e Department of Microbial Pathogenicity Mechanisms, Leibniz Institute for Natural Product Research and Infection Biology, Hans Knoell Institute, Jena, Germany; f Institute of Microbiology, Faculty of Biological Sciences, Friedrich Schiller University, Jena, Germany; g Unité Biologie et Pathogénicité Fongiques, Institut Pasteur, USC2019 INRA, Paris, France; h Université Paris-Saclay, CEA, CNRS, Institute for Integrative Biology of the Cell (I2BC), Gif-sur-Yvette, France; i Centre des Sciences du Goût et de l’Alimentation, AgroSup Dijon, CNRS, INRAE, Université Bourgogne Franche-Comté, Dijon, France; j Institut Necker Enfants-Malades (INEM), INSERM U1151-CNRS UMR 8253, Université de Paris, Paris, France

**Keywords:** *Candida albicans*, autophagy, epithelial cells, plasma membrane damage, lysosomal exocytosis

## Abstract

*Candida albicans* (*C. albicans*) is an opportunistic pathogen causing infections ranging from superficial to life-threatening disseminated infections. In a susceptible host, *C. albicans* is able to translocate through the gut barrier, promoting its dissemination into deeper organs. *C. albicans* hyphae can invade human epithelial cells by two well-documented mechanisms: epithelial-driven endocytosis and *C. albicans*-driven active penetration. One mechanism by which host cells protect themselves against intracellular *C. albicans* is termed autophagy. The protective role of autophagy during *C. albicans* infection has been investigated in myeloid cells; however, far less is known regarding the role of this process during the infection of epithelial cells. In the present study, we investigated the role of autophagy-related proteins during the infection of epithelial cells, including intestinal epithelial cells and gut explants, by *C. albicans*. Using cell imaging, we show that key molecular players of the autophagy machinery (LC3-II, PI3P, ATG16L1, and WIPI2) were recruited at *Candida* invasion sites. We deepened these observations by electron microscopy analyses that reveal the presence of autophagosomes in the vicinity of invading hyphae. Importantly, these events occur during active penetration of *C. albicans* into host cells and are associated with plasma membrane damage. In this context, we show that the autophagy-related key proteins ATG5 and ATG16L1 contribute to plasma membrane repair mediated by lysosomal exocytosis and participate in protecting epithelial cells against *C. albicans*-induced cell death. Our findings provide a novel mechanism by which epithelial cells, forming the first line of defense against *C. albicans* in the gut, can react to limit *C. albicans* invasion.

## Introduction

*Candida albicans* (*C. albicans*) is a normal inhabitant of the human commensal microbiota; however, it is also the most common fungal species responsible for opportunistic infections, causing candidiasis that can range from superficial to life-threatening invasive infections in debilitated patients.^1^Host-related conditions that confer high risk for *C. albicans*-associated infections include immune dysfunctions such as neutropenia, damage to the mucosal barrier, and dysbiosis of the resident bacterial microbiota.^2^ Invasive candidiasis occurs through three main stages including (i) the translocation of *C. albicans* through mucosal barriers in the bloodstream, (ii) its survival into the blood reservoir, and (iii) its escape from the bloodstream to secondarily infect deep-seated organs.^3^ Invasive candidiasis is associated with high mortality rates ranging from 20 to 49%.^1^Despite the fact that *C. albicans* colonizes various mucosal surfaces, such as the mouth or the vagina, most studies consider the gastrointestinal tract as the main portal of entry for *C. albicans* into the bloodstream.^3,4^ Indeed, molecular-based studies have identified the gut mycobiota as the main origin of disseminating *C. albicans* isolates into the blood.^5,6^

*C. albicans* is a polymorphic fungus that can grow in either yeast or hyphal morphology, and its ability to switch between these morphologies is considered as an important virulence trait.^7^ Additional morphotypes, including the gastrointestinally induced transition, the opaque^**a**/α^ and the gray morphotypes, may also support fungal adaptation to specific host niches.^7^ Beyond these morphological transitions, *C. albicans* promotes its pathogenicity not only by the expression of virulence factors comprising adhesins (*e.g*. Als or Hwp proteins families), invasins (*e.g*. Als3 and Ssa1), secreted hydrolases (*e.g*. the Sap protein family), and a toxin (candidalysin) but also by the formation of biofilm, sophisticated nutrient acquisition strategies, and a high metabolic flexibility.^1,8^

On the host side, innate defenses allow host cells to recognize and respond to fungal infections. The immune system continuously monitors the resident microbiota, and several mechanisms protect the gut from pathogenic microbes such as the production of antimicrobial peptides and immunoglobulin A, the secretion of mucus, and the presence of highly specialized phagocytic cells (macrophages, neutrophils, and dendritic cells).^9^ Detection of potentially pathogenic fungi and initiation of an innate immune response are achieved through Pattern Recognition Receptors (PRRs) expressed by various cell types in the gut, as exemplified by Dectin-1, which specifically recognizes β-glucans exposed at the fungal surface.^10^ Autophagy is an intracellular membrane-trafficking process that degrades cytosolic components by sequestrating them into double membrane vesicles termed autophagosomes that eventually fuse with lysosomes. Autophagy program activation requires the regulated and sequential involvement of more than 30 ATG (autophagy-related genes) proteins and notably includes two ubiquitin-like conjugation systems represented by the LC3 (ATG8) and ATG5/ATG12/ATG16L1 protein complexes.^11^ Early autophagosomal structures emanate from phosphatidyl-inositol-3-phosphate (PI3P)-enriched subdomains of the endoplasmic reticulum membrane (termed omegasomes) that allow the recruitment of PI3P-binding proteins such as WIPI2, which, in turn, promote autophagosomal membrane elongation and recruitment and stabilization of the ubiquitin-like conjugation systems.^12^ Of note, LC3 protein represents a standard marker for monitoring autophagy since the cytosolic form of LC3 (LC3-I) is conjugated to phosphatidylethanolamine (PE) on the surface of nascent autophagosomes. Autophagy is crucial to protect cells against various stresses and represents a key component of the innate immunity, especially by contributing to pathogen clearance and by modulating the inflammatory response in the gut.^13,14^ Autophagy has also been described as a protective mechanism against fungal infections.^15^ However, conflicting data exist on the role of autophagy *in vivo* during *C. albicans* infections. Indeed, it was suggested that mice deficient for autophagy (lacking functional *Atg5* or *Atg7* genes) in the myeloid cell lineage were more susceptible to *C. albicans* infections than wild-type mice,^16,17^ whereas no differences were observed in another related study.^18^ Most of the *in vitro* studies explored the role of autophagy during *C. albicans* infections in the context of immune cells.^15^ Studies using macrophages and dendritic cells have indeed demonstrated that *C. albicans* infections induce, in a Dectin-1 dependent manner, LC3-associated phagocytosis (LAP).^19^ LAP is a noncanonical form of autophagy, based on the recruitment of some components of the autophagy machinery to the phagosomal membrane that enhances acidification and killing efficiency of phagosomes.^20^ Of note, patients bearing a polymorphism, Y238X, in the Dectin-1 encoding gene (*CLEC7A*) displayed an increased oral and gastrointestinal colonization by *C. albicans*, compared to control patients.^21^

Much less is known regarding the role of autophagy during epithelial cell infection by *C. albicans*, with no study exploring its potential protective role in the gut context. Indeed, only two studies described autophagy activation during the infection of vaginal epithelial cells by *C. albicans*, an event that improved vaginal cell survival upon infection.^22,23^ Mucosal epithelial cells are at the frontline of host-microbe interactions and represent initial sentinels and responders to pathogens, thereby contributing to the efficiency of the innate immune response.^24^ In the gut, a single layer of epithelial cells confers a physical (*e.g*. tight junctions between cells) and a chemical (*e.g*. production of antimicrobial factors such as defensins) barrier between the host and its luminal microbiota. Autophagy has been described to contribute to multiple features of the protective role of epithelial cells, including tight junctions’ maintenance and cell death mitigation.^1,14^ Here, we explored in depth the role of autophagy and autophagy-related proteins in human epithelial cells during *C. albicans* infection, focusing on intestinal epithelial cells as the main portal of entry for *C. albicans*-disseminated infections. We showed that key autophagy-related proteins are recruited at *C. albicans* entry sites. This recruitment occurs only during active penetration by the fungi but not along the epithelial-driven endocytosis of *C. albicans* into epithelial cells. The recruitment of autophagy-related proteins to *C. albicans* entry sites is associated with plasma membrane damage and contributes to lysosomal membrane exocytosis, a process involved in plasma membrane repair. Finally, we showed that autophagy limits invasion and protects epithelial cells from *C. albicans*-induced cell death at early times of infection. This work provides new mechanistic insights into how host cells facing *C. albicans* invasion recruit components of the autophagic machinery.

## Results

### The autophagy-related protein LC3 is recruited at C. albicans entry sites

**Figure 1.**
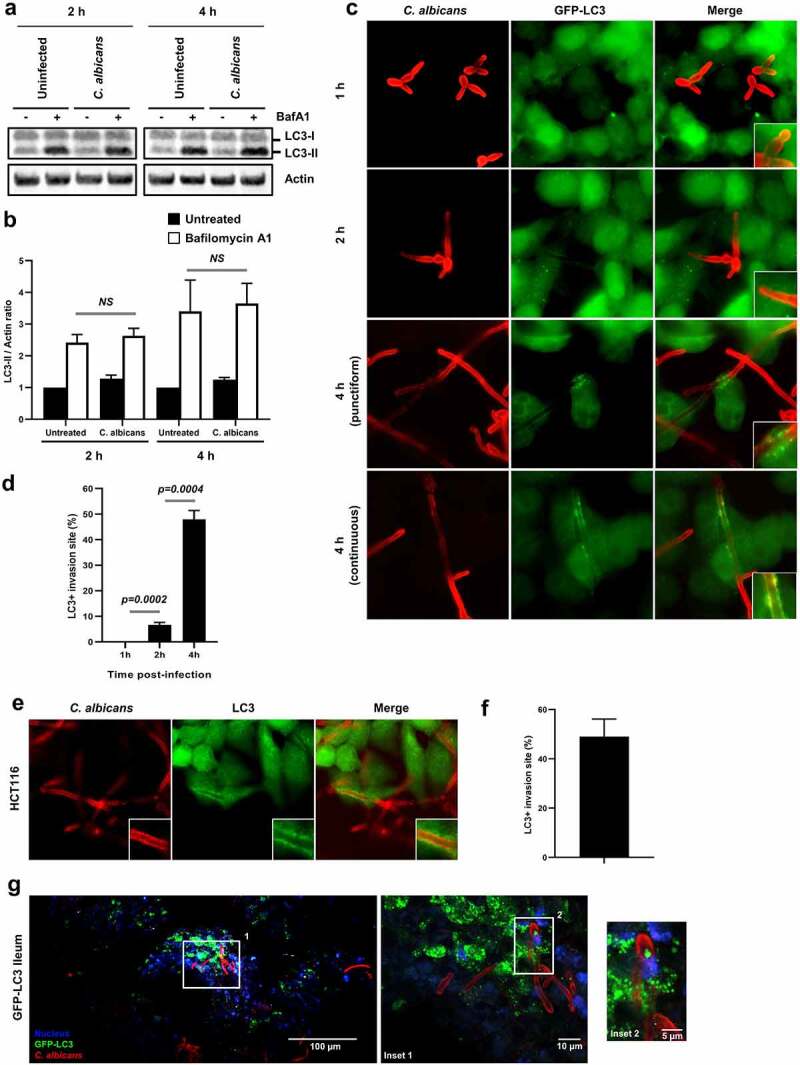
The autophagy-related protein LC3 is recruited at the C. albicans entry sites.

To determine whether a specific autophagic response is induced in host epithelial cells during infection by *C. albicans*, we first measured the autophagic flux by measuring LC3-II turnover by Western blot using the autophagy flux inhibitor Bafilomycin A1.^25^ During the activation of the autophagic process, cytosolic LC3 (termed LC3-I) is conjugated with PE and the amount of this lipidated form of LC3, termed LC3-II, correlates with the number of autophagosomes. Bafilomycin A1 prevents autophagosome-lysosome fusion and allows us to measure properly LC3-II accumulation upon stimulation. Immunoblot analysis of total protein extracts showed that infection of HeLa cells by *C. albicans* did not induce an increase in the steady state level of LC3-II at 2 and 4 h after infection, compared to uninfected cells (Figure 1a,b). Moreover, the inhibition of the autophagic flux by Bafilomycin A1 treatment did not allow us to visualize a significant increase of the LC3 lipidation upon infection. This apparent lack of marked global autophagy activation measured by immunoblot in the epithelial cell monolayer infected by *C. albicans* might be due to the fact that only a small subpopulation of host cells (around 10%, Sup Figure S1a) are invaded by the yeast at these time points. In order to explore autophagy activation only in infected cells, we used fluorescence microscopy to visualize the intracellular distribution of LC3. For this purpose, HeLa cells that stably express GFP-LC3 were infected for 1, 2, or 4 h with *C. albicans* (Figure 1c,d). At 1 and 2 h postinfection, only a few and isolated LC3 puncta, likely representing autophagosomes, were detected in the vicinity of invading *C. albicans* cells within epithelial cells, whereas at 4 h postinfection, a massive local recruitment of LC3 was observed in 47.9% of the *C. albicans* penetration sites (Figure 1d). This recruitment expressed as a “punctiform” to a “continuous” LC3 staining around the penetration sites likely corresponds to membrane-associated LC3 (*i.e*. LC3-II conjugated form) (Figure 1c). To confirm that LC3 observed at invasion sites is under its lipidated form, we transfected HeLa cells with the GFP-tagged lipidation defective mutant LC3 G120A^26^ and compared its recruitment with the wild-type GFP-LC3. Recruitment of this mutant form of LC3 is markedly reduced at *C. albicans* invasion sites, indicating that lipidation of LC3 is required for this cellular event (Sup Figure S1b,c). This finding was confirmed in cells expressing a dominant negative form of Atg4B (C74A), described to totally abrogate the lipidation of LC3,^27^ in which the recruitment of the endogenous LC3 was lost compared to control cells (Sup Figure S1d,e). We also used a special permeabilization protocol before cell fixation, which removes soluble LC3 and allows the staining of membrane-associated GFP-LC3. As shown in supplemental Figure 1e, LC3 recruitment at invasion sites confirmed the fact that LC3 was membrane-bound and might correspond to LC3-conjugated forms (Sup Figure S1f). The recruitment of endogenous LC3 was also observed in *C. albicans*-infected intestinal epithelial cells HCT116 (Figure 1e,f). Finally, ileal explant of GFP-LC3 transgenic mice was infected *ex vivo* by *C. albicans* for 4 h and LC3 accumulation was also noticed at entry sites of *C. albicans* hyphae into intestinal epithelial cells (Figure 1g). Altogether, these results indicate that *C. albicans* induces the recruitment of a component of the autophagy machinery, *i.e*. LC3, at its invasion sites *in vitro* and *ex vivo* in intestinal epithelial cells.

(**a**) Autophagy flux assay. HeLa cells were infected or not with *C. albicans* for 2 or 4 h, in the presence or absence of Bafilomycin A1 (BafA1) at 100 nM. Immunoblot analysis was performed using anti-LC3B and anti-actin antibodies. (**b**) The ratio of LC3-II signal relative to actin was measured and normalized to untreated cells without BafA1 (back bars: untreated, white bars: BafA1-treated). NS: not significant. (**c**) Representative images of *C. albicans*-infected GFP-LC3 HeLa cells after 1, 2, or 4 h infection. Samples were processed for *C. albicans* (red) and GFP-LC3 (green) staining. (**d**) Quantification of the percentage of GFP-LC3 + *C. albicans* invasion sites at 1, 2, or 4 h postinfection. Data are mean ± SEM of six independent experiments. (**e**) Representative images of *C. albicans*-infected HCT116 intestinal epithelial cells after 4 h of infection. Samples were processed for *C. albicans* (red) and endogenous LC3 (green) staining. (**f**) Quantification of the percentage of LC3 + *C. albicans* invasion sites at 4 h postinfection in HCT116 cells. Data are mean ± SEM of three independent experiments. (**g**) Representative images of ileal explants of GFP-LC3 mice infected for 4 h with *C. albicans*. Images were acquired using a two-photon excitation microscope. The white square indicates the inset area. Samples were processed for *C. albicans* (red), GFP-LC3 (green), and nuclei (blue) staining.

### Key components of the autophagy machinery are mobilized in the vicinity of C. albicans entry sites

**Figure 2.**
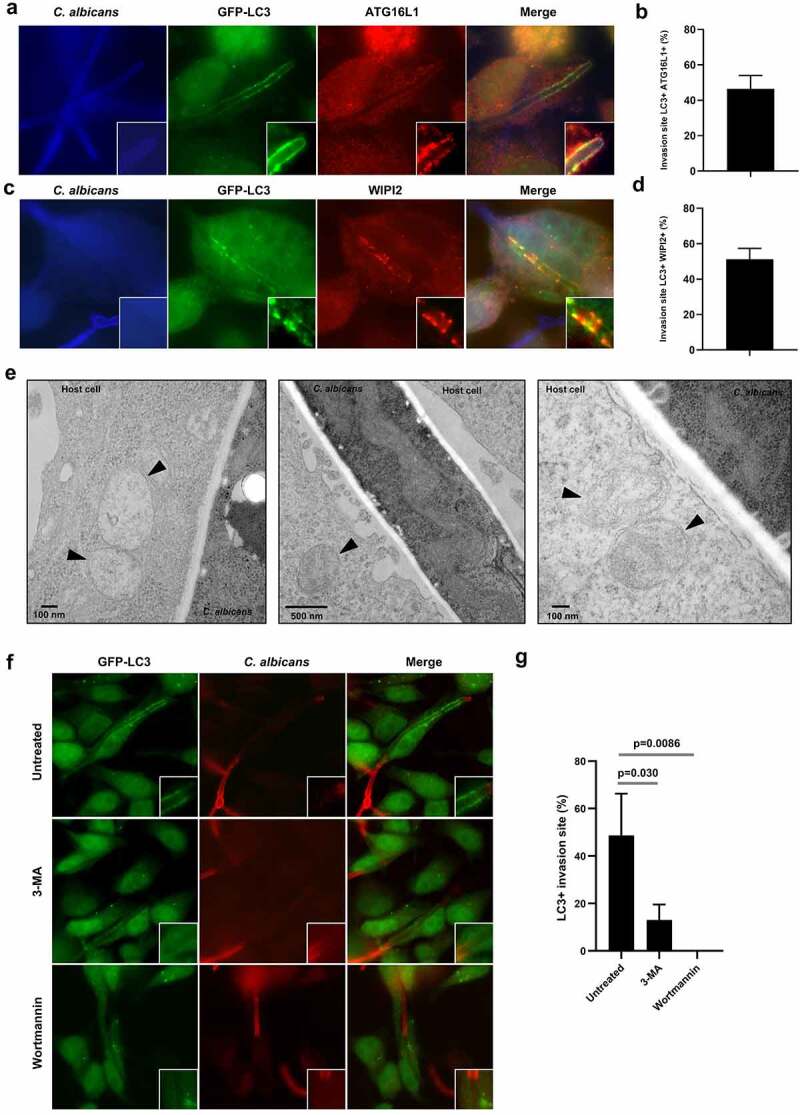
Key components of the autophagy machinery are mobilized in the vicinity of the C. albicans entry sites.

We next examined by immunofluorescence microscopy whether other key components of the autophagy machinery were recruited at the invasion sites of filamentous forms of *C. albicans* during infection of human epithelial cells. We first focus our attention on ATG16L1 and WIPI2, which participate in the initiation and elongation steps of the autophagic process. Interestingly, ATG16L1 and WIPI2 were markedly recruited at the *C. albicans* invasion sites at 4 h postinfection and partially colocalized with GFP-LC3 (Figure 2a–d). In addition, we observed the presence of cytosolic PI3P (the key lipid in autophagy initiation)-positive structures in the vicinity of *C. albicans* invasion sites (Sup Figure S2a).

(**a and c**) Representative images of *C. albicans*-infected GFP-LC3 HeLa cells after 4 h infection. Samples were processed for *C. albicans* (blue), GFP-LC3 (green), and (**a**) ATG16L1 (red) or (**c**) WIPI2 (red) staining. (**b and d**) Quantification of the percentage of (**b**) GFP-LC3+/ATG16L1+ or (**d**) GFP-LC3+/WIPI2 + *C. albicans* invasion sites at 4 h postinfection. Data are mean ± SEM of three independent experiments. (**e**) Ultrastructural analysis by transmission electron microscopy of HeLa cells infected with *C. albicans* for 4 h showing the presence of intracellular vacuoles with autophagosome features (indicated by black arrowheads) near *Candida* invasion sites. (**f**) Representative images of GFP-LC3 HeLa cells untreated or treated with the PI3K inhibitors 3-MA (5 mM) or Wortmannin (100 nM) and infected with *C. albicans* for 4 h. Samples were processed for *C. albicans* (red) and GFP-LC3 (green) staining. (**g**) Quantification of the percentage of GFP-LC3 + *C. albicans* invasion sites at 4 h postinfection. Data are mean ± SEM of three independent experiments.

The recruitment of ATG16L1 to the *C. albicans* invasion sites was dependent on its N‐terminal domain (Sup Figure S2b), which associates with ATG5 and ATG12 to promote LC3 lipidation (Sup Figure S2c) and its targeting to membranes during canonical and noncanonical autophagy.^28–30^ Indeed, an ATG16L1 protein truncated for the ATG5 binding domain (ATG16L1 ΔN), which lacks the ability to rescue LC3 lipidation in HeLa ATG16L1 KO cells (Sup Figure S2c), was no longer recruited at *C. albicans* invasion sites (Sup Figure S2d). By contrast, a mutant form of ATG16L1 lacking the C‐terminal WD40 domain (ATG16L1 ΔC) was able to partially rescue LC3 lipidation and was still mobilized at the *C. albicans* entry sites (Sup Figure S2c,d). In line with this observation, the large majority of LC3 + *C. albicans* invasion sites were positive for the phospho-ATG16L1 immunostaining (70.1%) (Sup Figure S2e,f). Interestingly, the Ser278 phosphorylated form of ATG16L1 has been recently identified as a marker of newly forming autophagosomes.^31^ Altogether, these observations suggest an active formation of autophagosomes at *C. albicans* invasion sites.

Given the important role of the Endoplasmic Reticulum (ER) membrane as an assembly platform in promoting the recruitment of autophagy-related proteins in the context of autophagosome biogenesis, especially at contact sites with the plasma membrane (PM),^32^ we next investigated whether ER markers also colocalized at *C. albicans* entry sites. We transfected HeLa cells with GFP-taggn tethering the ER to the PM^33^ and leading to the recruitment of key autophagy machinery components.^32^ Similarly, to LC3, GFP-E-Syt1 and GFP-E-Syt2 colocalized at the *C. albicans* penetration sites (Sup Figure S3a,b). Mobilization of ER-related membranes at *C. albicans* entry sites was confirmed by positive immunostaining for the endogenous ER-protein calreticulin in infected HeLa cells (Sup Figure S3c). By contrast, we were not able to notice at the invasion sites in HeLa cells an enrichment in mitochondria, an organelle also proposed as an assembly platform for autophagosome biogenesis,^34^ as indicated by immunostaining of the mitochondrial protein TOM20 (Sup Figure S3d).

The observed enrichment in these extended synaptotagmins 1 and 2 (E-Syt1 and E-Syt2) and two ER protein essentials in various autophagy-related proteins at the invasion sites of *C. albicans* prompted us to investigate the presence of autophagosomal vacuoles by transmission electron microscopy (TEM) in infected cells. We confirmed the presence of multiple intracellular vacuoles, presenting autophagosome features (*i.e*. double-membrane structures enclosing cytoplasmic content), close to the *C. albicans* entry sites in HeLa cells (Figure 2e), that corroborate, at least in part, the positive immunostaining for autophagy-related proteins observed at the invasion sites (see Figure 1c, 2a,c).

Since autophagy machinery activation relies on phosphoinositide 3-kinases (PI3Ks) class III signaling,^12^ we investigated the ability of PI3K inhibitors, *i.e*. 3-methyladenine (3-MA) and Wortmannin, to inhibit the recruitment of LC3 at *C. albicans* invasion sites. *C. albicans* infection concomitant treatment of cells with both inhibitors abrogated GFP-LC3 recruitment to the entry sites in GFP-LC3 HeLa cells (Figure 2f,g), supporting a role for the mobilization of the autophagy machinery in response to the stress induced by *C. albicans* penetration.

Finally, we measured local autophagosome maturation by infecting HeLa cells stably expressing a mRFP-GFP-LC3 construct with *C. albicans*. Autophagosome maturation was evaluated by discriminating early autophagic vacuoles (GFP+/mRFP+) from acidic autolysosomes (mRFP+/GFP-, since GFP is sensitive to acidic quenching).^25^ Although a slight increase in the proportion of mature autolysosomes was observed in the vicinity of *C. albicans* penetration sites in comparison to uninfected cells, the difference remained nonsignificant (Sup Figure S4a,b). Of note, the presence of some acidic compartments in the vicinity of *Candida* invasion sites was also confirmed by the accumulation of the lysotracker dye, a probe staining acidic organelles, in GFP-LC3 + *C. albicans* entry sites (Sup Figure S4c).

Altogether our results suggest that key players of the autophagy machinery are recruited at the invasion sites of *C. albicans* into host epithelial cells, along with the presence of intracellular vacuoles presenting autophagosome features. We hypothesize that stresses triggered at the host cell surface by the penetration of *C. albicans* filamentous forms mobilized the autophagy machinery.

### Autophagy machinery mobilization is associated with active penetration and not epithelial-driven endocytosis of C. albicans into epithelial cells

**Figure 3.**
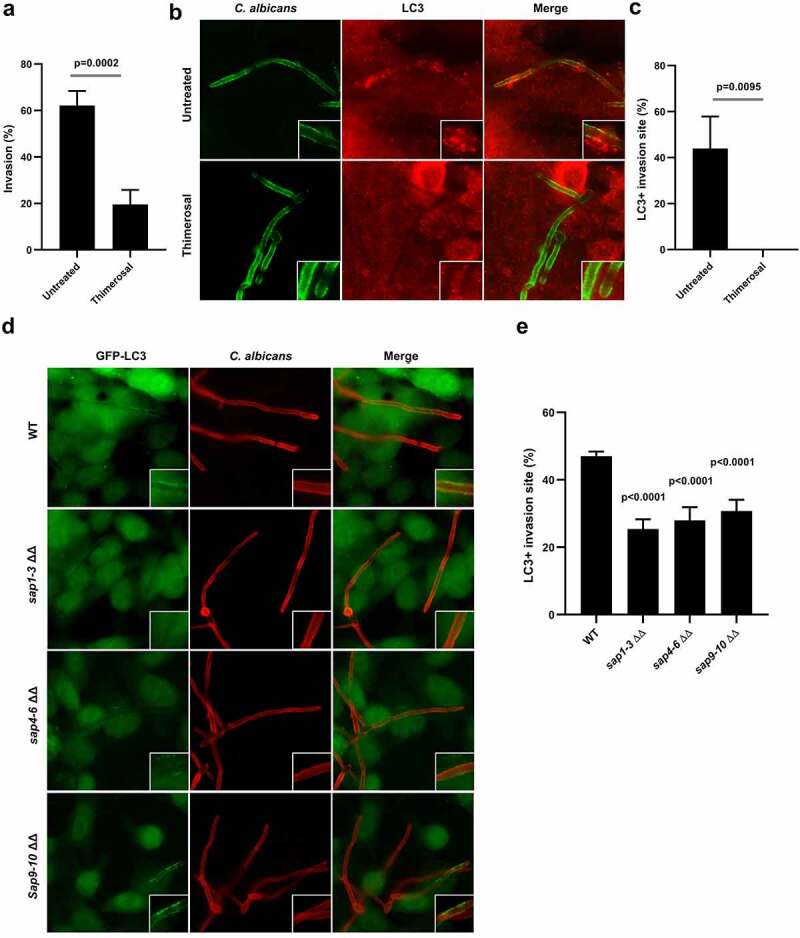
Autophagy machinery recruitment is linked to active penetration of C. albicans into epithelial cells.

*C. albicans* has developed defined strategies to invade epithelial cells, including two different well-described mechanisms that are dependent on the yeast-to-hyphae transition: (i) epithelial-driven endocytosis of hyphal forms and (ii) *C. albicans*-driven active penetration.^35–37^ To elucidate whether the recruitment of autophagy-related proteins at *C. albicans* entry sites during epithelial invasion was triggered by one of these two invasion mechanisms or both these invasion mechanisms, we used HeLa epithelial cells and blocked active penetration by treating *C. albicans* hyphae with thimerosal. This antifungal drug kills the fungus and thus selectively inhibits active penetration without affecting epithelial-driven endocytosis of the fungus.^38^ Pretreatment of *C. albicans* hyphae by thimerosal resulted in a total abrogation of *C. albicans* invasion in HeLa cells (Sup Figure S5a), confirming previous data that identified active penetration as the main invasion mechanism in HeLa cells during early phases of *C. albicans* invasion.^38^ Thus, no LC3 recruitment was observed at contact sites between thimerosal-treated *C. albicans* hyphae and HeLa cells (Sup Figure S5b). We performed additional experiments with the human intestinal epithelial cell line HCT116, a cell line that can be invaded by *C. albicans via* either active penetration or epithelial-driven endocytosis due to a low degree of differentiation.^39,40^ As a result, active penetration inhibition by pretreating *C. albicans* hyphae with thimerosal leads to a significant decrease in the percentage of *Candida* invasion (18.1%) compared to untreated hyphae (62.9%) (Figure 3a). Residual invasion (18.1%) of thimerosal-treated *C. albicans* was totally blocked by pretreatment of HCT116 cells with cytochalasin D, an endocytosis inhibitor, confirming that the residual invasion was related to epithelial-driven endocytosis of *C. albicans* (Sup Figure S5c). Collectively, these observations confirmed the occurrence of both invasion mechanisms in this cell line. Although thimerosal-treated *C. albicans* hyphae were still able to invade HCT116 cells through endocytosis, LC3 was no longer recruited at *C. albicans* invasion sites (Figure 3b,c). These results suggest that the recruitment of the autophagy-related protein LC3 at the plasma membrane is a cellular response specifically associated with active penetration of *C. albicans* hyphal forms rather than their endocytosis by epithelial cells.

(**a**) Percentage of invasion of untreated or thimerosal-pretreated *C. albicans* hyphae in HCT116 cells at 4 h postinfection. Each value is the mean of eight independent experiments ± SEM. (**b**) Representative images of untreated or thimerosal-pretreated *C. albicans* hyphae in HCT116 cells at 4 h postinfection. Samples were processed for *C. albicans* (green) and endogenous LC3 (red) staining. (**c**) Quantification of the percentage of LC3 + *C. albicans* invasion sites at 4 h postinfection. Data are mean ± SEM of four independent experiments. (**d**) Representative images of GFP-LC3 HeLa cells infected for 4 h by the wild-type strain of *C. albicans* (WT) or the corresponding *sap* 1/2/3 ΔΔ, *sap* 4/5/6 ΔΔ or *sap* 9/10 ΔΔ mutants. Samples were processed for *C. albicans* (red) and GFP-LC3 (green) staining. (**e**) Quantification of the percentage of LC3 + *C. albicans* invasion sites at 4 h postinfection. Data are mean ± SEM of four independent experiments.

To confirm these observations, GFP-LC3-expressing HeLa cells were infected with *C. albicans* mutants lacking genes encoding secreted aspartic proteases (Saps): *sap1-3*Δ/Δ, *sap4-6*Δ/Δ, and *sap9-10*Δ/Δ, that display a reduced ability to invade host cells by active penetration.^41–43^ For the three *SAP*-deficient mutants, a significant decrease in the percentage of LC3 + *C. albicans* invasion sites (*sap1-3*Δ/Δ: 25.4%, *sap4-6*Δ/Δ: 28.0%, and *sap9-10*Δ/Δ: 30.8%) was observed in comparison to cells infected with the wild-type strain (47.0%) (Figure 3d,e). The fact that *SAP*-deficient mutants, less able to invade host cells by active penetration, failed to recruit the autophagy-related protein LC3 at a level equivalent to that of the wild-type strain confirms the link between active penetration and the recruitment of autophagy-related proteins at *C. albicans* entry sites. Altogether, these results indicate that recruitment of components of the autophagy machinery is related to active penetration of *C. albicans* hyphal forms into host epithelial cells, but not to epithelial-driven endocytosis of the fungus.

### Recruitment of autophagy-related proteins to C. albicans entry sites is associated with plasma membrane damage

**Figure 4.**
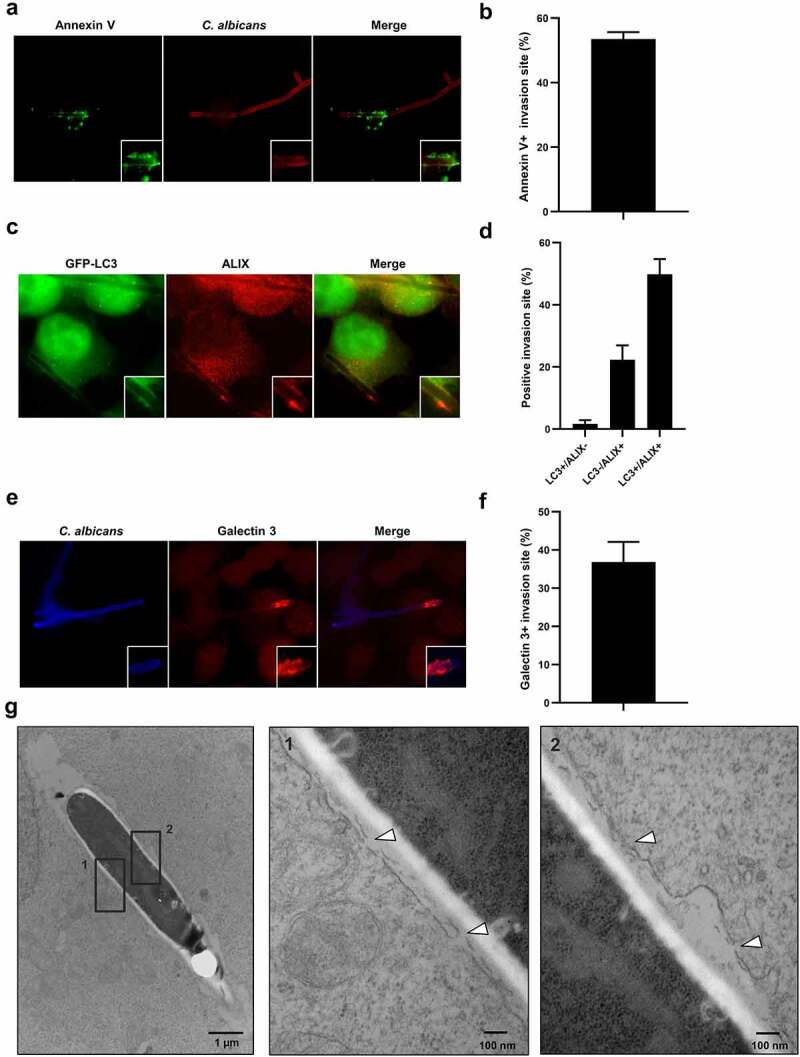
Recruitment of autophagy-related proteins to C. albicans entry sites is associated with plasma membrane damage.

Active penetration of *C. albicans* into host epithelial cells is likely to occur through molecular mechanisms combining the mechanical pressure exerted by elongating hyphae and the lytic activities of Saps and candidalysin.^1^ Both mechanisms can act as stressors that affect the host plasma membrane integrity. The recent literature described the contribution of autophagy-related proteins to the repair of damaged membranes, including the plasma membrane and endocytic vacuoles.^44–46^ Therefore, we hypothesized that the presence of autophagy-related proteins at the *C. albicans* penetration sites could be linked to plasma membrane damage response. To evaluate the presence of plasma membrane damage at LC3 + *C. albicans* penetration sites, we first examined the permeability of *C. albicans*-infected cells to the fluorescent Annexin V. This Ca^2^+-dependent phospholipid-binding protein has a high affinity for the phosphatidylserine phospholipid that is normally located on the cytoplasmic side of the plasma membrane and therefore not accessible for the binding to Annexin V. Thus, a positive Annexin V staining on cells indicates a loss of membrane integrity. About 53.5% of *C. albicans* invasion sites were positive for Annexin V staining at 4 h postinfection (Figure 4a,b), with a marked staining at the contact area between *C. albicans* and the host cell, suggesting the occurrence of plasma membrane damage. This was confirmed by the recruitment of ALIX (ALG-2-interacting protein X), an Endosomal Sorting Complexes Required For Transport (ESCRT) of binding protein described to be mobilized at the injured cell membrane,^47^ that colocalized with GFP-LC3 in 49.8% of *C. albicans* entry sites (Figure 4c,d). Additionally, membrane damage was also indicated by the recruitment of the endogenous Galectin-3 protein, colocalizing with GFP-LC3 in 36.9% of *C. albicans* entry sites (Figure 4e,f). Localization of Galectin-3 at *C. albicans* invasion sites was confirmed using a GFP-tagged version of this protein (Sup Figure S6a). Galectin is a protein recognizing galactose-containing glycans and well described for its recruitment at damaged membranes concomitantly to the ALIX protein.^48,49^ Interestingly, the Galectin-3 protein recruitment represents an early event in the recruitment of components of the autophagy machinery in various models of damaged endomembranes.^50,51^ Codistributions of ALIX and Galectin 3 with GFP-LC3 strongly suggest plasma membrane damage. As illustrated in Figure 4g, plasma membrane alterations were indeed observed by electron microscopy along the *C. albicans* invasion sites, with clear ruptures in the continuity of the membranes (Figure 4g). Of note, these alterations were not observed in HCT116 cells endocytosing *C. albicans* hyphae, displaying filopod-like structures and membrane ruffling on their surfaces (Sup Figure S6b). Collectively, these results strengthen the possible link existing between active penetration, plasma membrane damage, and the recruitment of autophagy-related proteins.

(**a**) Representative images of *C. albicans*-infected HeLa cells at 4 h postinfection. Samples were processed for *C. albicans* (red) and Annexin V (green) staining. (**b**) Quantification of the percentage of Annexin V + *C. albicans* invasion sites at 4 h postinfection. Data are mean ± SEM of six independent experiments. (**c**) Representative images of *C. albicans*-infected GFP-LC3 HeLa cells at 4 h postinfection. Samples were processed for ALIX (red) and GFP-LC3 (green) staining. (**d**) Quantification of the percentage of LC3+, ALIX+, or double positive *C. albicans* invasion sites at 4 h postinfection. Data are mean ± SEM of three independent experiments. (**e**) Representative images of *C. albicans*-infected HeLa cells at 4 h postinfection. Samples were processed for *C. albicans* (blue) and Galectin 3 (red) staining. (**f**) Quantification of the percentage of Galectin 3 + *C. albicans* invasion sites at 4 h postinfection. Data are mean ± SEM of three independent experiments. (**g**) Ultrastructural analysis by transmission electron microscopy of HeLa cells infected with *C. albicans* for 4 h, showing the presence of plasma membrane damage (indicated by white arrowheads) along the *C. albicans* invasion sites. Black squares indicate the two inset areas.

### The autophagy-related proteins, ATG16L1 and ATG5, contribute to the exocytosis of lysosomes at damaged plasma membranes

**Figure 5.**
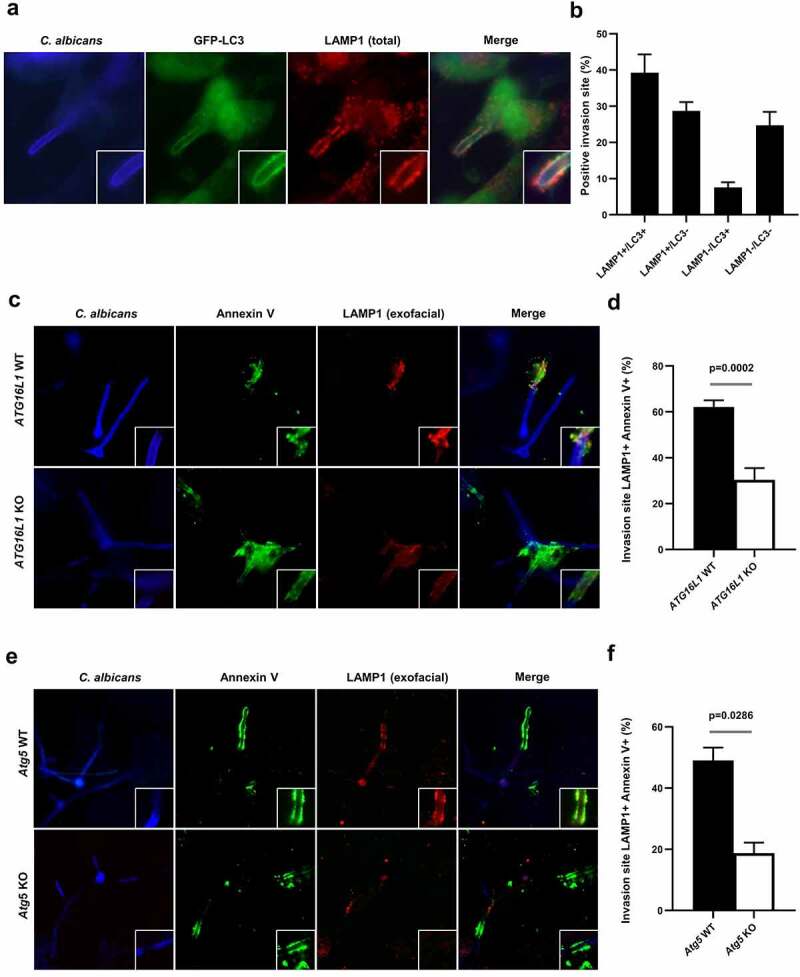
The autophagy-related proteins, ATG16L1 and ATG5, contribute to the exocytosis of lysosomes at the damaged plasma membrane.

In addition to the ESCRT machinery components, Ca^2+^ influx-driven exocytosis of lysosomes has been reported to contribute to membrane repair and their resealing.^52^ Recently, it has been proposed that some autophagy-related proteins are required to induce lysosomal membrane exocytosis in the context of cell injuries triggered by the bacterial pore forming toxins pneumolysin from *Streptococcus pneumoniae* and listeriolysin O from *Listeria monocytogenes*.^46^ We therefore investigated whether plasma membrane damage induced by active penetration of *C. albicans* hyphae correlated with mobilization of lysosomes, in association or not with the recruitment of autophagy-related proteins at damaged sites. For this purpose, we first analyzed the local recruitment of endogenous lysosomal-associated membrane protein 1 (LAMP1) at *C. albicans* invasion sites. By using immunofluorescence staining, we observed a marked recruitment of LAMP1 at *C. albicans* invasion sites (Figure 5a), which was confirmed by subcellular distribution of GFP-tag LAMP1, accumulating at most of the *C. albicans* invasion sites into epithelial cells (Sup Figure S7a). Similarly, lysosomal-associated membrane protein 2A (LAMP2A), another endogenous lysosomal-associated protein, localized at *C. albicans* invasion sites (Sup Figure S7b). Finally, LAMP1 codistributed with GFP-LC3 in 40% of the invasion sites, whereas 28.7% of entry sites were positive for LAMP1 only (Figure 5b). Collectively, these data suggest that plasma membrane damages promoted by active penetration of *C. albicans* hyphae into epithelial cells strongly mobilize lysosomal membranes, a phenomenon that is associated with the recruitment of autophagy-related proteins.

(**a**) Representative images of *C. albicans*-infected GFP-LC3 HeLa cells at 4 h postinfection. Samples were processed for *C. albicans* (blue), LAMP1 (red), and GFP-LC3 (green) staining. (**b**) Quantification of the percentage of GFP-LC3+, LAMP1+, or double positive *C. albicans* invasion sites at 4 h postinfection. Data are mean ± SEM of four independent experiments. (**c**) Representative images of *C. albicans*-infected HeLa cells depleted for *ATG16L1* (*ATG16L1* KO) or *C. albicans*-infected *ATG16L1* KO HeLa cells expressing the wild-type *ATG16L1* (*ATG16L1* WT) at 4 h postinfection. Samples were processed for *C. albicans* (blue), Annexin V (green), and exofacial LAMP1 (red) staining. (**d**) Quantification of the percentage of Annexin V + *C. albicans* also positive for LAMP1 staining at 4 h postinfection in *ATG16L1* WT (black bar) or *ATG16L1* KO (white bar) HeLa cells. Data are mean ± SEM of seven independent experiments. (**e**) Representative images of *C. albicans*-infected wild-type MEFs (*Atg5* WT) or *Atg5*-deficient MEFs (*Atg5* KO) at 4 h postinfection. Samples were processed for *C. albicans* (blue), Annexin V (green), and exofacial LAMP1 (red) staining. (**f**) Quantification of the percentage of Annexin V + *C. albicans* invasion sites also positive for LAMP1 staining at 4 h postinfection in *Atg5* WT (black bar) or *Atg5* KO (white bar) MEFs. Data are mean ± SEM of four independent experiments.

Next, to confirm lysosomal membrane mobilization at the surface of *C. albicans*-infected cells, we investigated LAMP1 protein delivery to the extracellular side of the plasma membrane as previously described.^46^ Without prior permeabilization, cells were stained alive on ice with an antibody recognizing an exofacial epitope of LAMP1, which allowed selective visualization of the pool of LAMP1 proteins exposed at the cell surface, as a consequence of lysosomal membrane exocytosis. The exofacial LAMP1 staining was only present along the penetration sites of the *C. albicans* hyphae (Figure 5c, upper panels), confirming the occurrence of lysosomal exocytosis following *C. albicans* invasion into epithelial cells. Exofacial LAMP1+ invasion sites were present in 62.2% of Annexin V+ epithelial cells, suggesting a strong association of lysosomal exocytosis with damaged plasma membranes (Figure 5c,d). Finally, since lysosomal membrane exocytosis is a calcium-triggered process, cells were treated with the calcium-chelating agent BAPTA-AM before infection to prevent elevation in the intracellular calcium concentration. As a consequence, we observed a significant decrease in the percentage of LC3 and LAMP1 signals at *C. albicans* invasion sites in BAPTA-AM-treated cells (8.0%) compared to untreated cells (45.1%) (Sup Figure S7c,d), indicating that calcium signaling contributes to the recruitment of LC3 and LAMP1 to the invasion sites.

Interestingly, a significant decrease in lysosomal exocytosis was observed in CRISPR/Cas9-mediated *ATG16L1* gene knock-out HeLa cells as only 30.4% of *C. albicans* invasion sites were positive for exofacial LAMP1 in these cells staining compared to 62.2% in HeLa cells expressing WT *ATG16L1* (Figure 5c, lower panels, and D). Similar results were obtained using murine *Atg5*-deficient cells, with a significant decrease in the percentage of exofacial LAMP1 + *C. albicans* invasion sites in *Atg5* knock-out cells (18.8%) compared to wild-type cells (49%) (Figure 5e,f). Altogether, these results showed that (i) lysosomal membrane exocytosis occurs at *C. albicans* penetration sites in epithelial cells and (ii) autophagy-related proteins, including ATG16L1 and ATG5, contribute to this process.

### The recruitment of autophagy-related proteins limits invasion and protects cells from C. albicans-induced epithelial cell death

**Figure 6.**
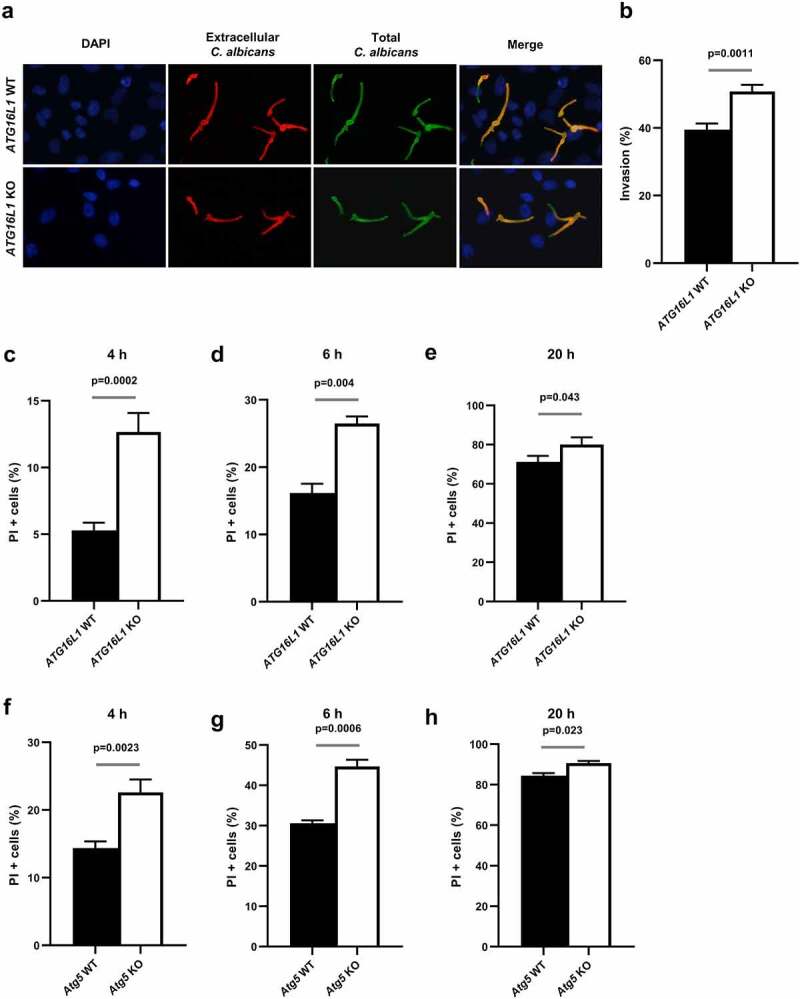
The recruitment of autophagy-related proteins limits invasion and protects cells from C. albicans-induced epithelial cell death.

Since autophagy-related proteins are actively recruited during active penetration of *C. albicans* and seem to contribute to plasma membrane repair by favoring lysosomal exocytosis, we questioned whether autophagy-related proteins are associated with the frequency of invasion events. We therefore, compared the percentage of *C. albicans* invasion between wild-type and the autophagy-deficient *ATG16L1* KO HeLa cells. A differential staining protocol allowing discrimination between extracellular (red + green) and intracellular (only green) fungal hyphae was performed to measure the percentage of internalized hyphae^35^(Figure 6a). After 4 h of infection, invasion of the *ATG16L1*-deficient cells by *C. albicans* was slightly, but significantly increased (50.8%) compared to control cells (39.5%) (Figure 6a,b). Following invasion, the infection of epithelial cells is characterized by a loss of epithelial integrity associated with the death of host cells invaded by *C. albicans* invasion.^53^ This cell death is potentially attributed to plasma membrane damage caused by mechanical elongation of invading hyphae and the expression of *C. albicans* virulence factors such as the Saps proteases and/or the candidalysin toxin.^38,53,54^ Therefore, we tested whether ATG16L1-deficient cells were more sensitive to *C. albicans*-induced cell death. Viability of epithelial cells was measured by staining cells with propidium iodide (PI), a cell-death marker. At early times postinfection (4 and 6 h), higher percentage of cell death was observed in ATG16L1-deficient cells compared to wild-type cells (Figure 6c,d). At later time postinfection (20 h), percentage of dead cells increased dramatically compared to early time points (4 and 6 h) in ATG16L1 wild-type or deficient cells, with a slight, but yet significant difference between the two genotypes (Figure 6e). Very similar results were obtained when comparing *C. albicans*-induced cell death in Atg5-deficient cells with the corresponding wild-type cells (Figure 6f–h). These results strongly suggest that autophagy-related proteins (ATG16L1 and ATG5) participate in cells’ protection against *C. albicans*-induced cell death at early time points postinfection. However, this protective role was restricted to early time points and less effective at later stages of the infection process.

(**a**) Representative images of *C. albicans* invasion assay performed in *ATG16L1* WT or *ATG16L1* KO HeLa cells at 4 h postinfection. Samples were processed for nuclei (blue), extracellular *C. albicans* (red), and total *C. albicans* (green) stainings. (**b**) Percentage of *C. albicans* invasion in *ATG16L1* WT (black bar) or *ATG16L1* KO (white bar) HeLa cells. Data are mean ± SEM of eight independent experiments. (**c, d, and e**) Percentage of cell death as measured by propidium iodide incorporation (PI+ cells) in *ATG16L1* WT (black bar) or *ATG16L1* KO (white bar) HeLa cells infected by *C. albicans* for (**c**) 4 h, (**d)** 6 h, or (**e**) 20 h postinfection. Data are mean ± SEM of six independent experiments. (**f, g, and h**) Percentage of cell death as measured by propidium iodide incorporation (PI+ cells) in *Atg5* WT MEFs (black bar) or *Atg5* KO MEFs (white bar) infected by *C. albicans* for (**f**) 4 h, (**g**) 6 h, or (**h**) 20 h postinfection. Data are mean ± SEM of seven independent experiments.

## Discussion

**Figure 7.**
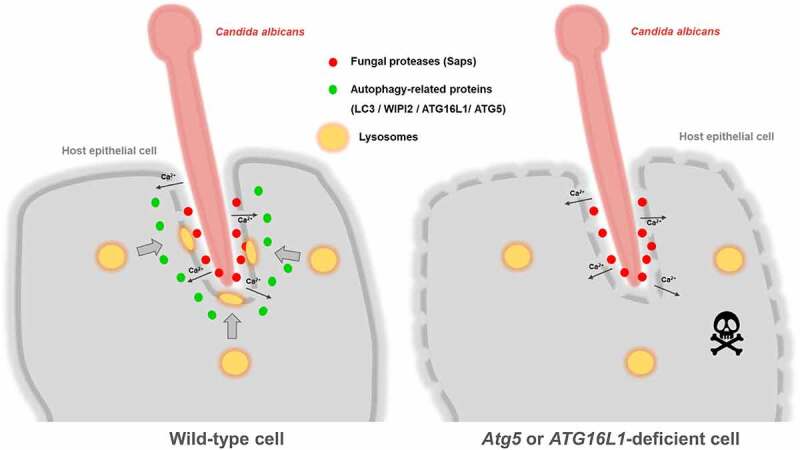
Schematic representation of the hypothetical role of autophagy-related proteins following active penetration of epithelial cells by C. albicans.

A growing number of studies described autophagy and autophagy-related proteins as key players in epithelial response against pathogens.^55–59^ In this study, we explored the involvement of the autophagy machinery during host epithelial cell response to *C. albicans* infections. We showed that several autophagy-related proteins (LC3, ATG16L1, and WIPI2) are recruited to *C. albicans* invasion sites during infection of epithelial cells, including intestinal epithelial cells (cell line and gut explants). This phenotype was associated with autophagosome biogenesis close to invasion sites since early markers of autophagosome formation (PI3P, WIPI2, and phospho-ATG16L1) are detected in the vicinity of these locations. These events only occur during active penetration of *C. albicans*, and not during induced endocytosis, and are associated with plasma membrane damage. Finally, we demonstrate that the recruitment of the autophagy-related proteins ATG5 and ATG16L1 contributes to lysosomal exocytosis during *C. albicans* invasion, a mechanism involved in plasma membrane repair,^60^ and protects epithelial cells against *C. albicans*-induced cell death at early stages of infection (schematic representation in Figure 7). To our knowledge, this is the first detailed characterization of autophagy-related proteins implication during invasion of host intestinal epithelial cells by the fungal pathogen *C. albicans*.

Based on the results of our study, we hypothesize that in wild-type cells (left side), active penetration of *C. albicans* hyphae and secretion of the fungal proteases Saps (red dots) induce plasma membrane damage, causing influx of Ca^2+^ (black arrows) into infected cells. Several key players of the autophagy machinery (illustrated by green dots), such as LC3, WIPI 2, ATG16L1, and ATG5, are recruited at *C. albicans* invasion sites and contribute to the exocytosis of lysosomes (yellow rounds), thereby limiting the deleterious effects of *C. albicans* invasion into host cells. In *Atg5*- or *ATG16L1*-deficient cells (right side), the exocytosis of lysosomes is dramatically reduced and cells are more sensitive to *C. albicans*-induced cell death, especially at early time postinfection.

While the role of autophagy and autophagy-related proteins is well described in bacterial or viral infections, far less is known about their roles in response to eukaryotic pathogens, including fungi, especially in the case of *C. albicans* that is only partially internalized into host cells. Indeed, *C. albicans* hyphae are able to penetrate epithelial cells without being fully internalized into host cells, even at late time postinfection.^1^ Thus, in contrast to *C. albicans* yeast cells phagocytosed by macrophages or dendritic cell, *C. albicans* hyphae are rarely fully engulfed by nonprofessional phagocytic host cells and do not end up in full enclosed endocytic or autophagic vacuoles.^17,61^ This is also in contrast to observations made for some other eukaryotic pathogens. For example, *Plasmodium* sp. or *Toxoplasma gondii* or *Leishmania amazonensis* can be fully internalized into autophagosomes or at least into vacuoles harboring autophagy markers in hepatocytes or macrophages.^62–64^ Regarding fungal pathogens, a number of studies have reported *Cryptococcus neoformans* or *Aspergillus fumigatus* as the inducer of LC3-associated phagocytosis (LAP) in infected macrophages or dendritic cells, known to be specialized phagocytic cells.^65^ This noncanonical form of autophagy required the recruitment of a part of the proteins of the autophagic machinery (including the PI3KC3 complex, ATG3, ATG5, ATG7, ATG12, and ATG16L1) to microorganisms that are engulfed within a single membrane vacuole (phagosome or endosome).^66^ We here report that even if *C. albicans* is rarely fully internalized within host epithelial cells’ cytoplasm, the membranes at invasion sites are massively decorated by several autophagy-related proteins. Additionally, features of active autophagosome biogenesis (as indicated by the presence of LC3 under its conjugated form and positive staining for PI3P, phospho-ATG16L1, and WIPI2) were monitored in close association with this subcellular area, suggesting that the autophagy machinery is mobilized in the immediate vicinity of the plasma membrane following the external stress applied by *C. albicans* penetration. Our findings in an infectious context are in line with studies demonstrating that the cytosolic side of plasma membrane is an important subcellular compartment for autophagosome biogenesis.^32,67^ However, since many reports describe a wide range of autophagy-independent roles for ATG proteins, especially in membrane dynamics events,^68,69^ we cannot rule out the possibility that a part of the ATG proteins recruited at *C. albicans* entry sites fulfills additional functions, independent of their role in canonical autophagosome biogenesis, in particular by contributing to lysosomal exocytosis.

Contribution of some autophagy-related proteins (ATG16L1, ATG5, and ATG12) in lysosomal exocytosis was recently highlighted in the context of host cell infection by the Gram-positive pathogenic bacterium *Listeria monocytogenes*.^46^ Cells lacking these ATG proteins were unable to trigger lysosomal membrane exocytosis for the repair of membrane damage induced by bacterial pore-forming toxins (LLO and PLY). As a result, an increase in sensitivity of cells to bacterial toxins was observed.^46^ Interestingly, we report herein similar observations showing that plasma membrane damage induced by *C. albicans* correlates with (i) a strong decrease in lysosomal membrane exocytosis as observed in *ATG16L1*- and *Atg5*-deficient cells and (ii) an increase sensitivity of these deficient cells to *C. albicans*-mediated cell death. This plasma membrane damage might result from the mechanical stress applied by the fungal active penetration in association with secreted factors (including Saps) already reported to alter the host plasma membrane.^1^

In the present study, a massive recruitment of ATG16L1, WIPI2, and LC3 (including its conjugated form LC3-II) occurred at *C. albicans* invasion sites presenting plasma membrane damage that was not observed in plasma membrane damage induced by bacterial pore-forming toxins.^46^ We hypothesize that, in the case of *C. albicans*, the plasma membrane damage is focused in a limited area of the plasma membrane (around the active penetration sites) at some stages of infection. This damage is associated with a strong mechanical deformation of the plasma membrane, a stimulus favorable to the recruitment of proteins involved in membrane dynamics regulation.^70^ This last point offers the intriguing possibility for a role of autophagy machinery, or at least for some autophagy-related proteins, in response to mechanical deformation of the plasma membrane. Since autophagy and its associated proteins are well described as components of stress responsive pathways for mammalian cells, we could assume that autophagy-related structures and/or proteins could play a role following mechanical stress sensed by the plasma membrane. To our knowledge, there is no study describing in depth such a role for autophagy, but it has been recently shown that shear stress in epithelial cells induced by fluid flow is sufficient to mobilize the autophagy machinery.^71^ Further investigations are thus needed to clearly understand to which extent the autophagy machinery contributes to the response of the plasma membrane to mechanical deformation. In addition, since several hallmarks of autophagosome biogenesis were present at *C. albicans* invasion sites, we could not exclude that autophagic vesicles contribute directly to plasma membrane repair by providing a pool of membrane available for membrane resealing.

In conclusion, the data presented here describe the contribution of the autophagy machinery at the plasma membrane in response to invasion of *C. albicans* into epithelial cells. The recruitment of autophagy-related proteins is associated with markers of autophagosomes biogenesis, suggesting that *de novo* synthesis of autophagic vacuoles occurs in response to *C. albicans* active penetration into host cells. We assume that this recruitment of ATG proteins is not fully dedicated to canonical autophagosome biogenesis, but participates in broader host cell response mechanisms aimed at coping with *C. albicans*-imposed mechanical (plasma membrane deformation) and chemical (fungal proteases and toxins) stresses and the subsequent plasma membrane damage. This hypothesis is in perfect line with the growing literature placing autophagy-related proteins as key players of membrane dynamics.^68^ Functionally, this recruitment of ATG proteins plays a protective role for infected cells by contributing to membrane repair mediated by lysosomal exocytosis and by limiting host cell death at early time postinfection. An interesting challenge in the future will be to explore whether this massive mobilization of lysosomes and vacuoles presenting features of autophagosome at the plasma membrane could release specific cargoes in the extracellular compartment able to limit *C. albicans* invasion by affecting its integrity, representing thereby an additional protective mechanism to ensure intestinal barrier integrity. Interestingly, such a role of autophagy-related proteins in orchestrating the release of lysosomal material in the extracellular compartment has been described few years ago in the context of osteoclastic bone resorption.^72^ Some autophagy-related proteins (ATG5, ATG7, ATG4B, and LC3) have been shown to be recruited to the bone-apposed plasma membrane of osteoclasts and to contribute to the exocytosis of lysosomes that release hydrolases and acidify the bone microenvironment to digest the organic matrix of bone.^72^ Our study, by describing similar findings in the context of *C. albicans*-apposed plasma membranes of epithelial cells, paves the way for future investigations to decipher whether lysosomes released in this situation by host cells could be detrimental to *C. albicans* integrity, thereby protecting the host cell.

## Materials and methods

### Cell lines

Cell lines used in this study are listed in Table 1. HeLa and HCT116 were grown in DMEM with GlutaMax (DMEM-GlutaMax with phenol red, Gibco, 31966–021) supplemented with 10% fetal bovine serum (FBS, PanBiotech, P30-8100) without antibiotics, at 37°C and 5% CO_2_. GFP-LC3-HeLa (kindly provided by Mathias Faure), mRFP-GFP-LC3-HeLa (kindly provided by David C. Rubinsztein), ATG16L1 WT and KO HeLa cells (both kindly provided by Xavier Ramnik), and HFF and Strawberry-ATG4B-C74A-HFF cells were maintained under the same conditions. The *Atg5* Tet-Off MEF m5-7 cells (kindly provided by Noboru Mizushima) were maintained in DMEM with GlutaMax containing 10% FBS with doxycycline (Dox, 20 ng/mL) for one week to fully suppress *Atg5* expression.^77^ All cell lines were routinely tested for mycoplasma contamination using the PCR Mycoplasma Test Kit II (PromoKine).

**Table 1.** Cell lines used in this study

Cell lines	Description	Applications	Origin/Reference
HeLa	Human epithelial cells (cervix)	Immunoblot immunofluorescence invasion electron microscopy	ATCC
HCT116	Human epithelial cells (colon)	Immunofluorescence invasion electron microscopy	ATCC
GFP-LC3-HeLa	HeLa cells stably expressing GFP-tagged wild-type LC3	Immunofluorescence	^73^
mRFP-GFP-LC3-HeLa	HeLa cells stably expressing mRFP and GFP-tagged wild-type LC3	Immunofluorescence	^74^
ATG16L1 KO HeLa	HeLa cells knocked-out for the *ATG16L1* gene using theCRISPR-Cas9 system	Immunofluorescence invasion cell death assay	^75^
ATG16L1 WT HeLa	HeLa cells knocked-out for the *ATG16L1* gene and complemented with WT ATG16L1	Immunofluorescence invasion cell death assay	^75^
HFF	Human foreskin fibroblasts	Immunofluorescence	^76^
Strawberry-ATG4B-C74A-HFF	HFF stably expressing the Strawberry-tagged ATG4B C74A dominant negative	Immunofluorescence	^76^
*Atg5* Tet-Off MEF m5-7	Mouse embryonic fibroblasts with a tetracycline-regulated expression of Atg5	Immunofluorescence cell death assay	^77^

### Plasmid transfection

HeLa cells or ATG16L1 KO HeLa cells were transiently transfected for 24 h using Lipofectamine 2000 (Thermo Fischer Scientific) with the indicated plasmid (listed in Table 2 and detailed in the figure legend) according to the manufacturer’s protocol. During and after transfection, cells were maintained in DMEM with GlutaMax supplemented with 10% FBS, without antibiotics, at 37°C and 5% CO_2_.

**Table 2.** Plasmids used in this study

Plasmids	Description	Origin/Reference
pcDNA-mCherry-ATG16L1 WT	mCherry-tagged wild-type ATG16L1 (autophagy-related protein)	^78^
pcDNA-mCherry-ATG16L1 ΔC	mCherry-tagged ATG16L1 lacking the C-terminal domain	^78^
pcDNA-mCherry-ATG16L1 ΔN	mCherry-tagged ATG16L1 lacking the N-terminal domain	^78^
LAMP1-mGFP	GFP-tagged LAMP1 (lysosomal-associated protein)	Addgene (34831)
pEGFP-E-Syt1	GFP-tagged E-Syt1 (ER-associated protein)	^32^
pEGFP-E-Syt2	GFP-tagged E-Syt2 (ER-associated protein)	^32^
pEGFP-LC3	GFP-tagged wild-type LC3 (autophagy-related protein)	^79^
pEGFP-LC3 G120A	GFP-tagged lipidation defective mutant of LC3	^79^
pEGFP-Galectin 3	GFP-tagged Galectin 3 (Lectin binding to the damaged membrane)	^80^

### C. albicans strains and infection

Strains used in this study are listed in Table 3. The *C. albicans* strain SC5314 was used for all assays except for the assay in Figure 3 (panels D and E), where the *C. albicans* strain BWP17-CIp30 was used as the parental strains of the *SAP*-deficient mutants. Fungi were maintained on solid Sabouraud dextrose agar 2%. For experiments, *C. albicans* was grown in YPD liquid medium (1% yeast extract, 2% peptone, 2% dextrose, and Fischer Bioreagent) at 37°C overnight (ON), in a shaking incubator. Suspension was adjusted at an optical density (OD) of 600 nm equal to 0.3 in fresh YPD medium and incubated for 2 h more for log phase. *C. albicans* yeast forms were then harvested, sonicated, counted, and adjusted to the desired concentration in Dulbecco’s Modified Eagle’s Medium (DMEM, Gibco). For infection, indicated cell lines were infected with 5.10^4^ log phase *C. albicans* cells for 1 to 4 h and maintained in DMEM with GlutaMax at 37°C and 5% CO_2_.

**Table 3.** Candida strains used in this study

Strain name	N° interne	Acronym	Control strain	Genotype
SC5314		WT		Clinical isolate from London Mycological Reference Laboratory
BWP17-CIp30	M2251	Parental strain		RPS/rps1::(URA3 HIS1 ARG4)
∆*sap9*/∆*sap10* + pCIp10	M1429		BWP17-CIp30	*sap10*∆::hisG/*sap10*∆::hisG *sap9*∆::hisG/*sap9*∆::hisG rps::URA3
∆*sap1/2/3* + pCIp10	M1630		BWP17-CIp30	*sap1*∆::hisG/*sap1*∆::hisG *sap2*∆::hisG/*sap2*∆::hisG *sap3*∆::hisG/*sap3*∆::hisG rps::URA3
∆*sap4/5/6* + pCIp10	M1632		BWP17-CIp30	*sap6*∆::hisG/*sap6*∆::hisG *sap4*∆::hisG/*sap4*∆::hisG *sap5*∆::hisG/*sap5*∆::hisG rps::URA3

For hyphal growth, 10^6^ yeast forms were seeded on 6-well plates (Costar, VWR) for 4 h in RPMI-1640 medium (Gibco, Life Technologies) at 37°C, 5% CO_2_. Hyphae were then washed three times in PBS, scraped, and counted. For chemical inactivation, *C. albicans* yeast forms were incubated in 0.04% Thimerosal-PBS solution for 1 h at room temperature (RT). *Candida* was then washed in PBS and counted.

### Antibodies and reagents

For immunoblotting, we used the following primary antibodies: rabbit polyclonal anti-LC3B (7543) and rabbit polyclonal anti-Actin (A2066), purchased from Sigma, and the secondary antibody anti-rabbit IRDye 680RD Goat anti-Rabbit IgG (926–68071) purchased from Li-Cor. For immunofluorescence experiments, we used the primary antibodies described below. Rabbit monoclonal anti-ATG16L1 (D6D5) and rabbit monoclonal anti-Alix-1 (92880S) were purchased from Cell Signaling Technology. Rabbit polyclonal anti-LC3B (7543) was purchased from Sigma. Rabbit polyclonal anti-*Candida albicans* (BP-1006) was purchased from Origene. Rabbit polyclonal anti-Calreticulin (10292–1) was purchased from Proteintech. Rabbit polyclonal anti-LAMP2A (ab18528) and rabbit monoclonal anti-phospho-ATG16L1 (Ser278) (EPR19016) were purchased from Abcam. Mouse monoclonal anti-LAMP1 (H4A3) was purchased from DSHB Iowa. Mouse monoclonal anti-WIPI 2 (2A2) was purchased from Millipore. Mouse monoclonal anti-TOM20 (F-10) was purchased from Santa Cruz Biotechnology. Mouse monoclonal anti-Galectin-3 (MAB11541) was purchased from R&D systems. Mouse monoclonal anti-anti β-D-glucans (400–2) was purchased from Biosupplies. Fluorescent secondary antibodies: Alexa Fluor-conjugated anti-rabbit IgG −350, −488, and −568 and Alexa Fluor-conjugated anti-mouse IgG −350, −488, −568 were purchased from Invitrogen (A11046, A11034, A11036, A31552, A11001, A11004, and 1/500), and FITC-conjugated anti-GST antibody was purchased from Rockland.

DAPI for labeling nuclei, thimerosal (T5125, 0.04%) for fungal inactivation, cytochalasine D (500 nM) for endocytosis inhibition, and wortmannin (W1628, 100 nM) and 3-MA (M9281, 5 mM) for inhibiting autophagy were purchased from Sigma. BAPTA-AM (10 μM, 196419, Millipore) was used as a cell-permeant Ca^2+^ chelator. The acidotropic dye Lysotracker probe DND-99 (100 nM) was purchased from Invitrogen. Bafilomycin A1 (100 nM) for inhibiting autophagy flux was purchased from LC laboratories. FITC-Annexin V (PK-CA707-30017) for detecting plasma membrane damage was purchased from Promokine. Propidium Iodide (#638) for staining dead cells was purchased from Immunochemistry technologies.

### Infection of ileal explants from GFP-LC3 mice

Transgenic GFP-LC3 mice were provided by the “RIKEN BioResource Research Center” (RBRC00806; depositor: Noboru Mizushima,^81,82^ Mice were housed at INRAE facilities, Dijon, France (accreditation number: C21 231 010 EA). All procedures involving animal experimentation were carried out according to the European guidelines for the care and use of laboratory animals. The ileum from GFP-LC3 homozygous mice were collected in prewarmed (37°C) DMEM medium with 1% antibiotics (penicillin/streptomycin) (Eurobio). The ileum was flushed with DMEM to remove fecal contents and opened longitudinally with scissors. Once washed in fresh DMEM medium with 1% antibiotics, the ileum was dissected into 5 mm per 5 mm explants with a sterile scalpel blade and explants were placed with the luminal side up into 6-well culture plates containing prewarmed DMEM medium with 1% antibiotics (2 mL per well). Explants were infected with 5.10^5^ log phase yeast of *C. albicans* for 4 h at 37°C and 5% CO_2_.

### Autophagy flux assay

HeLa cells were pretreated with Bafilomycin A1 at 100 nM for 30 min prior to infection with *C. albicans*. Bafilomycin A1 was then maintained in the cell culture medium for 2 or 4 h before total protein extraction.

### Immunoblot analysis

Whole-cell protein extracts from HeLa cells were prepared from cell monolayer by adding directly 200 µL of 1.25× Laemmli sample buffer. Cell lysates were disrupted by sonication for 5 min, and proteins were denatured by heating at 95°C for 5 min. Protein extracts were clarified by a centrifugation step for 10 min at 4 000 g (room temperature). Equal amounts of protein were subjected to SDS-PAGE (4–15% mini-Protean precast protein gel, Bio-Rad), transferred on the nitrocellulose membrane (Trans-blot turbo, Bio-rad), and then immunoblotted using the indicated primary antibodies. Anti-rabbit and anti-mouse antibodies conjugated with IR800 or IR680 dyes were used as secondary antibodies, and the infrared signal was integrated using an infrared imaging system (LI-COR Odyssey). The band intensities were calculated using the software associated with the Odyssey system (Image studio).

### Immunostaining

After fungal infection, cells were washed once with PBS and fixed with 4% paraformaldehyde (PFA) in PBS (0.01 M, pH7.4) for 15 min at room temperature (RT) and washed 3 times in PBS. Cells were permeabilized in 0.1% Triton X-100/PBS for 15 min at RT. Cells were then blocked by 10% goat serum in PBS for 30 min. Cells were incubated with the indicated primary antibodies for 2 h in PBS at RT. Cells were then washed three times with PBS and incubated with the secondary Alexa Fluor-conjugated antibodies for 45 min at RT. Cells were washed three times with PBS and mounted in fluorescence mounting medium (ProLong Gold Antifade, Thermo Fisher Scientific). All images were acquired using a fluorescence microscope (Axiovision Zeiss).

For the staining of membrane-associated GFP-LC3 in HeLa cells, cells were washed once with PBS and gently dipped for 3 s in cold permeabilization buffer (TritonX-100 0.1% in 100 mM KCl, 2 mM MgCl_2_, 1 mM CaCl_2_ and 1 mM Hepes, pH 6.9). Then, cells were washed once with PBS and fixed with 4% PFA as described above.

For PI3P staining in GFP-LC3 HeLa cells, cells were washed once with PBS and fixed with 4% in PBS (0.01 M, pH7.4) for 15 min at RT, washed 3 times in PBS and incubated for 1 h with purified FYVE-FYVEGST-recombinant protein (20 μg/mL final concentration), washed with PBS, and labeled with a FITC-conjugated anti-GST antibody as previously described.^83^

For ileal explants staining, tissues were fixed overnight at 4°C in 4% PFA in PBS (0.01 M, pH7.4). After washing in PBS, explants were permeated/saturated in 1% BSA-PBS, 0.1% Triton X-100, and 0.05% tween 20, for 1 h at room temperature, then incubated overnight at 4°C with rabbit polyclonal anti-*C. albicans* (BP-1006, Origene), and diluted in saturating buffer. Tissues were rinsed three times in PBS and incubated with the goat anti-rabbit secondary antibody (Alexa fluor 594, A11012, Thermo Fisher scientific) in saturating buffer, for 1 h at room temperature. After washing, tissues were mounted in antifading solution. Cell nuclei were visualized with DAPI. Images were acquired on a two-photon excitation microscope (Nikon A1-MP, DImaCell platform).

### Exofacial LAMP1 and Annexin V staining

Exofacial LAMP1 staining was performed as previously described.^46^ After infection, cells (ATG16L1 WT/KO HeLa cells or *Atg5* Tet-Off MEF m5-7) were washed once with cold HBSS with calcium (HBSS+) and then incubated in HBSS+ containing 10% goat serum for 20 min on ice with 1% (v/v) LAMP1 antibody (H4A3), 5% (v/v) FITC-Annexin V to visualize exofacial phosphatidylserine, and *C. albicans* antibody. Cells were then washed with HBSS+ and fixed in 4% PFA (in PBS+) for 15 min at RT. Cells were washed in HBSS+ and incubated in secondary antibodies Alexa Fluor 568 and 350 conjugated for 30 min at RT. Cells were finally washed in HBSS+, and coverslips were mounted on slides using fluorescence mounting medium.

### Invasion assay

Invasion of *C. albicans* to epithelial cells (ATG16L1 WT/KO HeLa or HCT116 cells) quantification was determined as previously described.^35^ Briefly, epithelial cells were grown to confluence for 48 h on a coverslip and then infected in DMEM-containing GlutaMax with 5.10^4^ log phase yeast of *C. albicans* for 1 to 4 h in a humidified incubator (37°C/5% CO2). Cell monolayers were rinsed three times with PBS and fixed with 4% PFA for 10 min at RT. The adherent part of *C. albicans* (extracellular) was stained for 1 h with the anti-*Candida* antibody, washed in PBS three times, and then incubated with the Alexa Fluor 568-conjugated secondary antibody. After three washing in PBS, cells were permeabilized with 0.1% Triton X-100/PBS for 15 min. All parts of yeasts (extracellular and intracellular) were stained with the anti-*Candida* antibody for 1 hour and subsequently with Alexa Fluor 488-conjugated secondary antibody. Coverslips were rinsed three times in PBS and mounted and observed with a fluorescence microscope (Axiovision Zeiss). The percentage of invading *C. albicans* was determined by dividing the number of totally or partially internalized *C. albicans* by the total number of adherent *C. albicans*. At least 100 *C. albicans* were counted on each coverslip.

### Transmission electron microscopy (TEM)

Experiments were carried out in the CellImaP core facility. Cells (HeLa or HCT116), grown on Thermanox coverslips, were fixed for 1 h at 4°C in 4% paraformaldehyde and 2.5% of glutaraldehyde in Sorensen phosphate buffer (0.1 mM, pH 7,3). After fixation, samples were washed by Sorensen phosphate buffer. The postfixation treatment was realized with 1% osmium tetroxide at RT for 1 h. Dehydration and resin impregnation of the samples were performed manually: dehydration was done by increasing degrees of ethyl alcohol (50°, 70°, 95°, and 100°) and substitution was done by three absolute ethanol: Embed-812 resin mixtures and impregnation in Embed-812 resin. The polymerization of samples was performed with a mixture Embed-812: 3% BDMA in the gelatin capsule maintained for 48 h at 60°C. Blocks were cut on an ultramicrotome, and slices (thickness of 60 nm) were deposited on copper/palladium grids. After drying, grids were contrasted with uranyl acetate and lead citrate. TEM observations of cells were realized at the Dimacell imaging facility (Dijon, France) on a HITACHI H-7500 operating at 80 kV.

### Cytotoxicity assay

After infection, cells (ATG16L1 WT/KO HeLa cells or *Atg5* Tet-Off MEF m5-7) were washed with PBS and incubated with 0.1% propidium iodide in PBS for 15 min on ice. Cells were then fixed with 4% PFA for 15 min at RT and stained for *C. albicans* as described above. The percentage of cytotoxicity was determined in fungal invaded cells.

### Statistical analysis

All experiments were performed at least 3 times. Statistical analyses were performed using GraphPad Prism software (GraphPad Software Inc., San Diego, CA, USA). The nonparametric Mann and Whitney t test was used to compare results between conditions. The *p*-values ≤ 0.05 were considered as statistically significant.

## Supplementary Material

Supplemental Material

## Data Availability

The data that support the findings of this study are available from the corresponding authors upon reasonable request.

## Abbreviations

ALIX: ALG-2-interacting protein X

Als: Agglutinin-like sequence

ATG: Autophagy-related gene

*C. albicans: Candida albicans*

ER: Endoplasmic Reticulum

E-Syt1-2: Extended synaptotagmins 1-2

ESCRT: Endosomal Sorting Complexes Required For Transport

GFP: Green fluorescent protein

Hwp: Hyphal wall protein

LAMP1: Lysosomal-associated membrane protein 1

LC3: Microtubule-associated proteins 1A/1B light chain 3

LLO: Listeriolysin O

PI3K: Phosphoinositide 3-kinase

PI(3)P: Phosphatidylinositol-3-phosphate

PLY: Pneumolysin

PM: Plasma membrane

RFP: Red fluorescent protein

Sap: Secreted aspartyl proteinases

TEM: Transmission electron microscopy

TOM20: Translocase of outer membrane 20

WIPI2: WD repeat domain phosphoinositide-interacting protein 2
