# Supplementary material for: Membrane protective role of autophagic machinery during infection of epithelial cells by Candida albicans
Source: Gut Microbes. 2022 Jan 27;14(1):2004798. doi: 10.1080/19490976.2021.2004798 (PMC8803057; doi:10.1080/19490976.2021.2004798)
Supplement: Supplemental Material [file KGMI_A_2004798_SM7022.zip › Supplementary information/Lapaquette_et_al_supplemental_figures.docx]

***
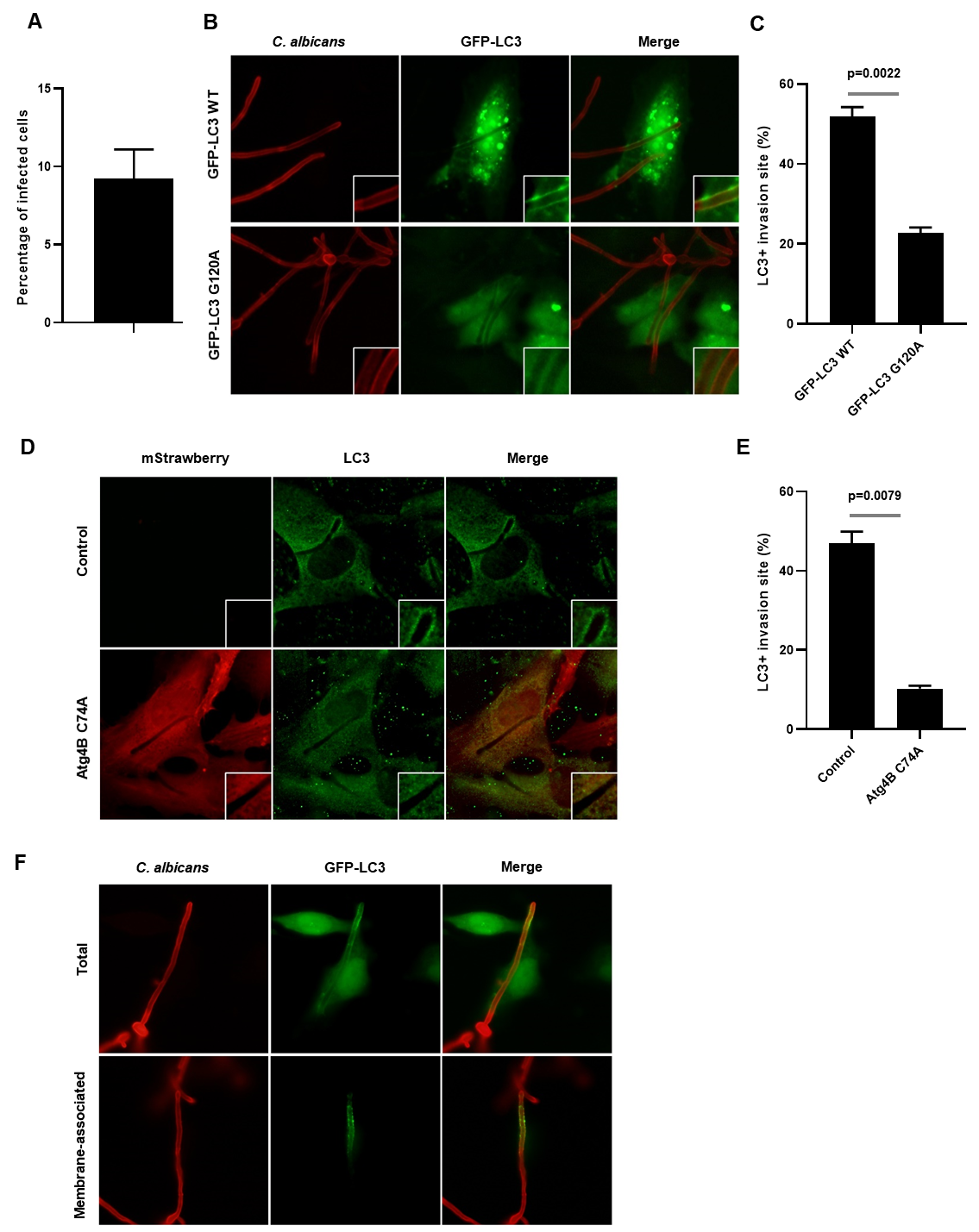
 Supplementary Figure 1:***

(**A**) Percentage of Hela cells infected by *C. albicans* at 4 h post-infection. Data are mean +/- SEM of six independent experiments. (**B**) Representative images of HeLa cells transfected with GFP-LC3 WT (upper panels) or lipidation defective mutant GFP-LC3 G120A (lower panels) and infected for 4 h with *C. albicans*. Samples were processed for *C. albicans* (red) and GFP-LC3 (green) staining. (**C**) Percentage of GFP-LC3+ *C. albicans* invasion sites at 4 h post-infection. Data are mean +/- SEM of six independent experiments. (**D**) Representative images of *C. albicans*-infected control fibroblast (upper panels) or fibroblast expressing the Strawberry-tagged Atg4B mutant C74A (lower panels) after a 4 h infection. Samples were processed for endogenous LC3 (green) and Atg4B (red). (**E**) Percentage of LC3+ *C. albicans* invasion sites at 4 h post-infection. Data are mean +/- SEM of five independent experiments. (**F**) Representative images of *C. albicans*-infected GFP-LC3 HeLa cells after 4 h infection. Samples were processed for GFP-LC3 (green) and *C. albicans* (red) after a regular fixation conserving total protein in cells (upper panels) or after a permeabilization step before fixation enabling to visualize membrane-associated proteins (lower panels).


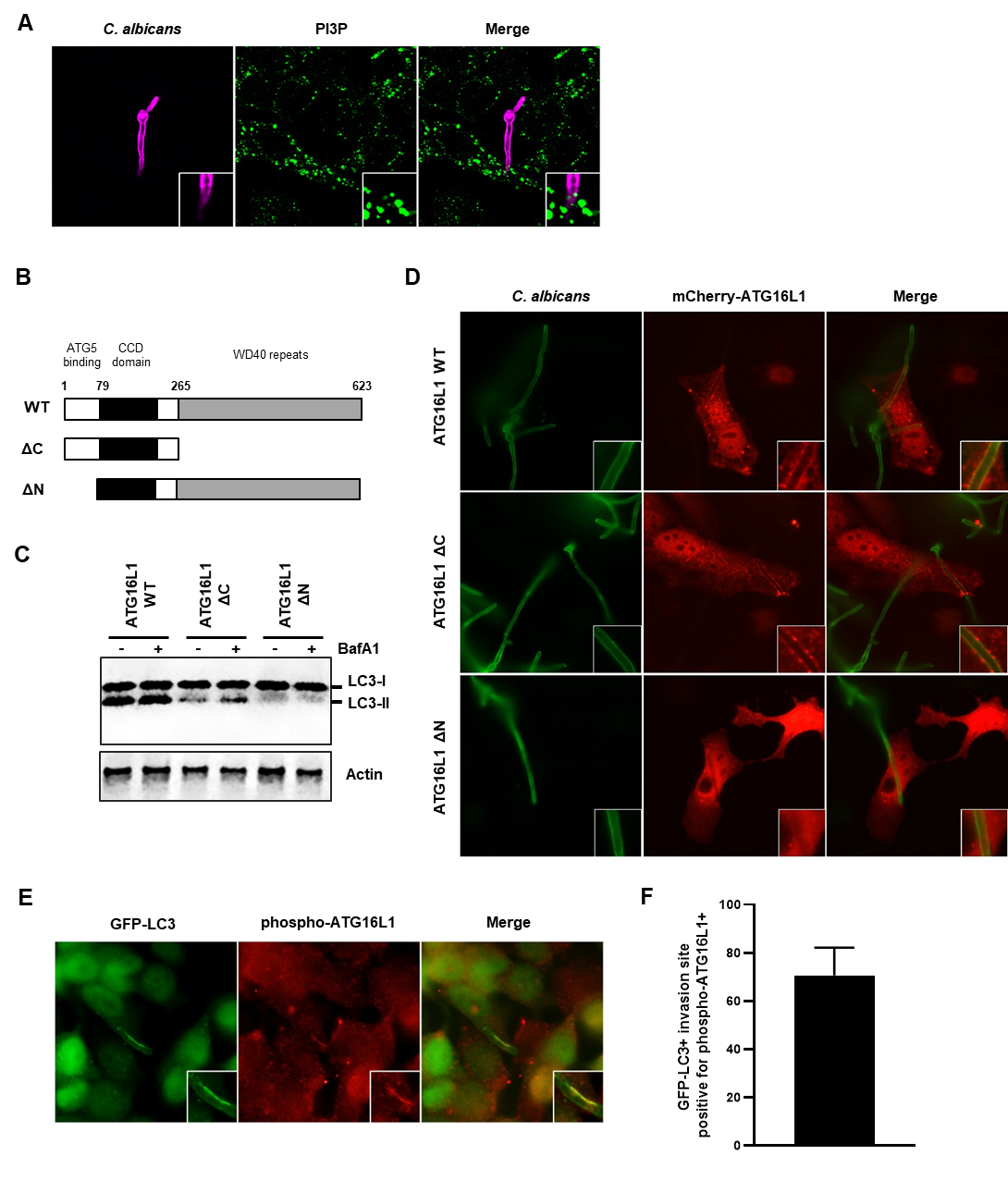


***Supplementary Figure 2:***

(**A**) Representative images of *C. albicans*-infected GFP-LC3 HeLa cells at 2 h post-infection. Samples were processed for *C. albicans* (purple) and PI3P (green) staining. (**B**) Diagram of full-length (WT) and deletion constructs (ΔC lacking the WD40 repeats domain and ΔN lacking the ATG5 binding domain) of ATG16L1 used in this study. (**C**) ATG16L1 KO HeLa cells were transfected with mCherry-tagged ATG16L1 constructs described in B and treated for 2 h or not with Bafilomycin A1 (BafA1) at 100 nM to assess autophagy flux. Immunoblotting was performed using anti-LC3 and anti-Actin antibodies. (**D**) Representative images of ATG16L1 KO HeLa cells transfected with mCherry-tagged ATG16L1 constructs (described in B) and infected for 4 h with *C. albicans*. Samples were processed for *C. albicans* (green) and mCherry-ATG16L1 (red) staining. (**E**) Representative images of *C. albicans*-infected GFP-LC3 HeLa cells after 4 h infection. Samples were processed for GFP-LC3 (green) and phospho-ATG16L1 (red). (**F**) Percentage of GFP-LC3+ *C. albicans* invasion sites also positive for phospho-ATG16L1 staining at 4 h post-infection. Data are mean +/- SEM of three independent experiments.


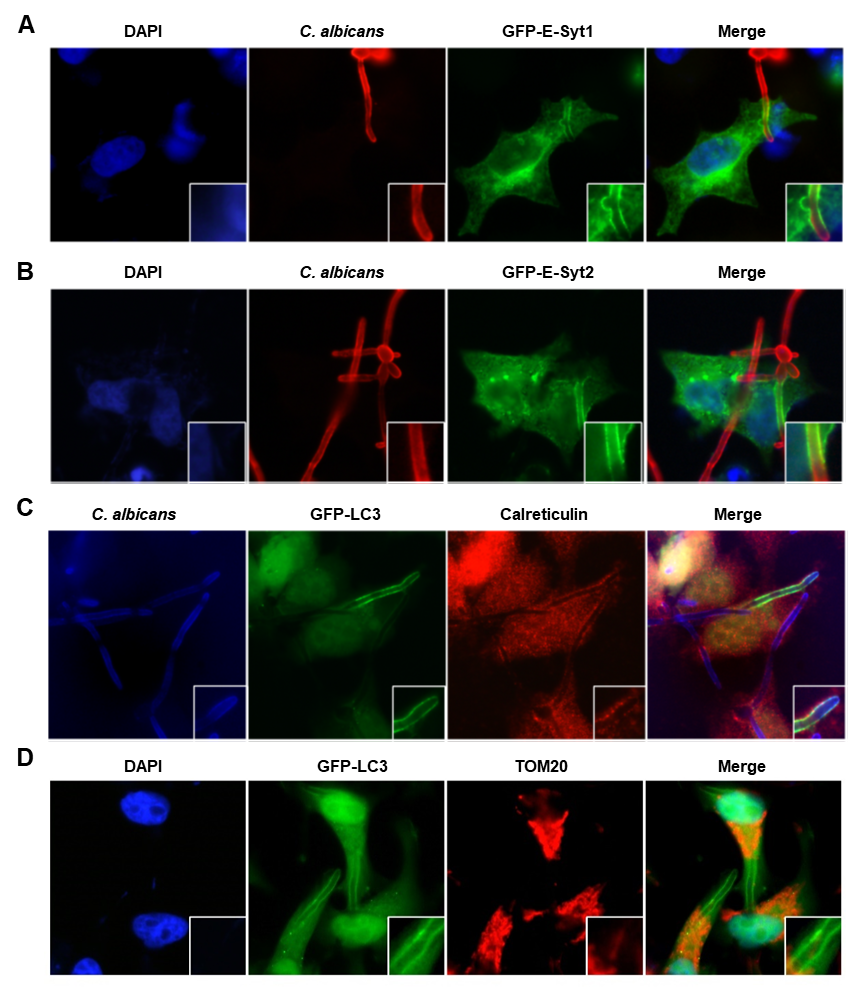


***Supplementary Figure 3:***

(**A,B**) Representative images of HeLa cells transfected with (**A**) GFP-E-Syt1 or (**B**) GFP-E-Syt2 and infected for 4 h with *C. albicans*. Samples were processed for nuclei (blue), GFP-E-Syts (green) and *C. albicans* (red). (**C, D**) Representative images of *C. albicans*-infected GFP-LC3 HeLa cells at 4 h post-infection. Samples were processed for GFP-LC3 (green) and (**C**) calreticulin (red) or (**D**) TOM20 (red).


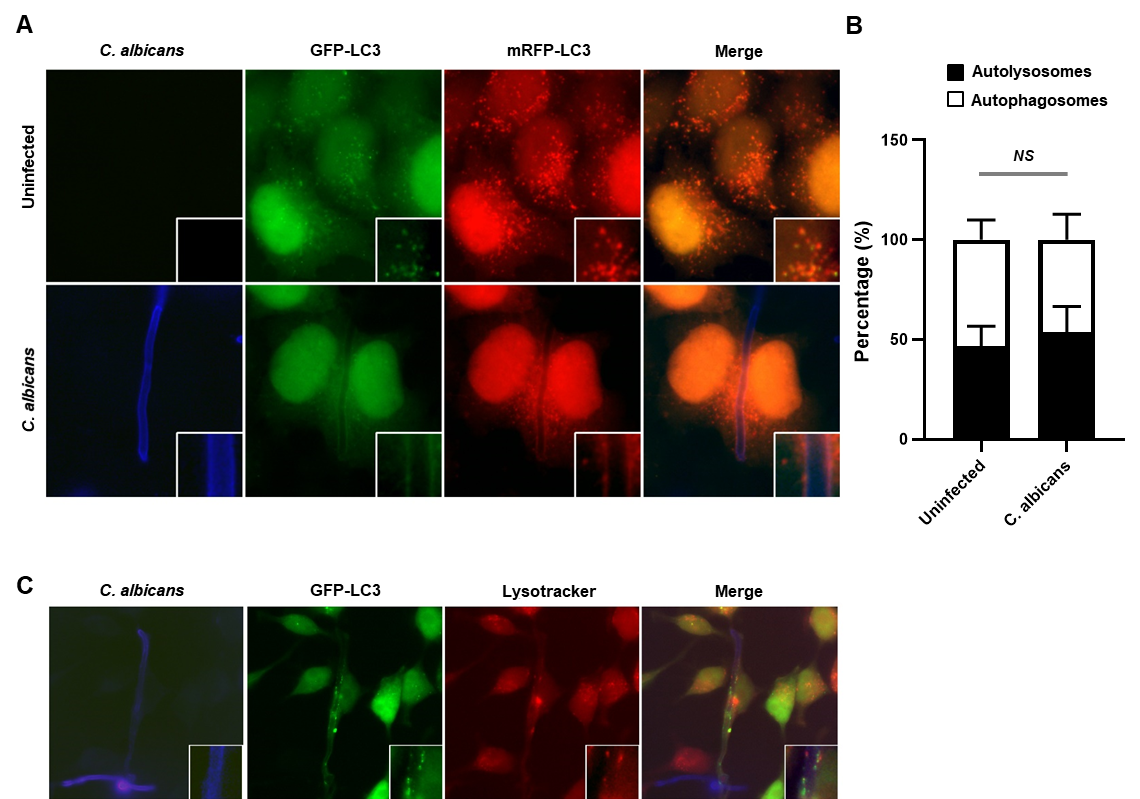


***Supplementary Figure 4:***

(**A**) Representative images of uninfected or *C. albicans*-infected mRFP-GFP-LC3-HeLa cells at 4 h post-infection. Samples were processed for *C. albicans* (blue) and mRFP-GFP-LC3 (red and green) staining. (**B**) Quantification of the number of autophagosomes (RFP+ GFP+ dots) and autolysosomes (RFP+GFP− dots) per cell. Results are expressed as the relative proportion of autolysosomes (black bars) and autophagosomes (white bars). Each value is the mean of three independent experiments ± SEM. (**C**) Representative images of *C. albicans*-infected GFP-LC3 HeLa cells after 4 h infection. Samples were processed for GFP-LC3 (green) and the acidotropic dye lysotracker (red).


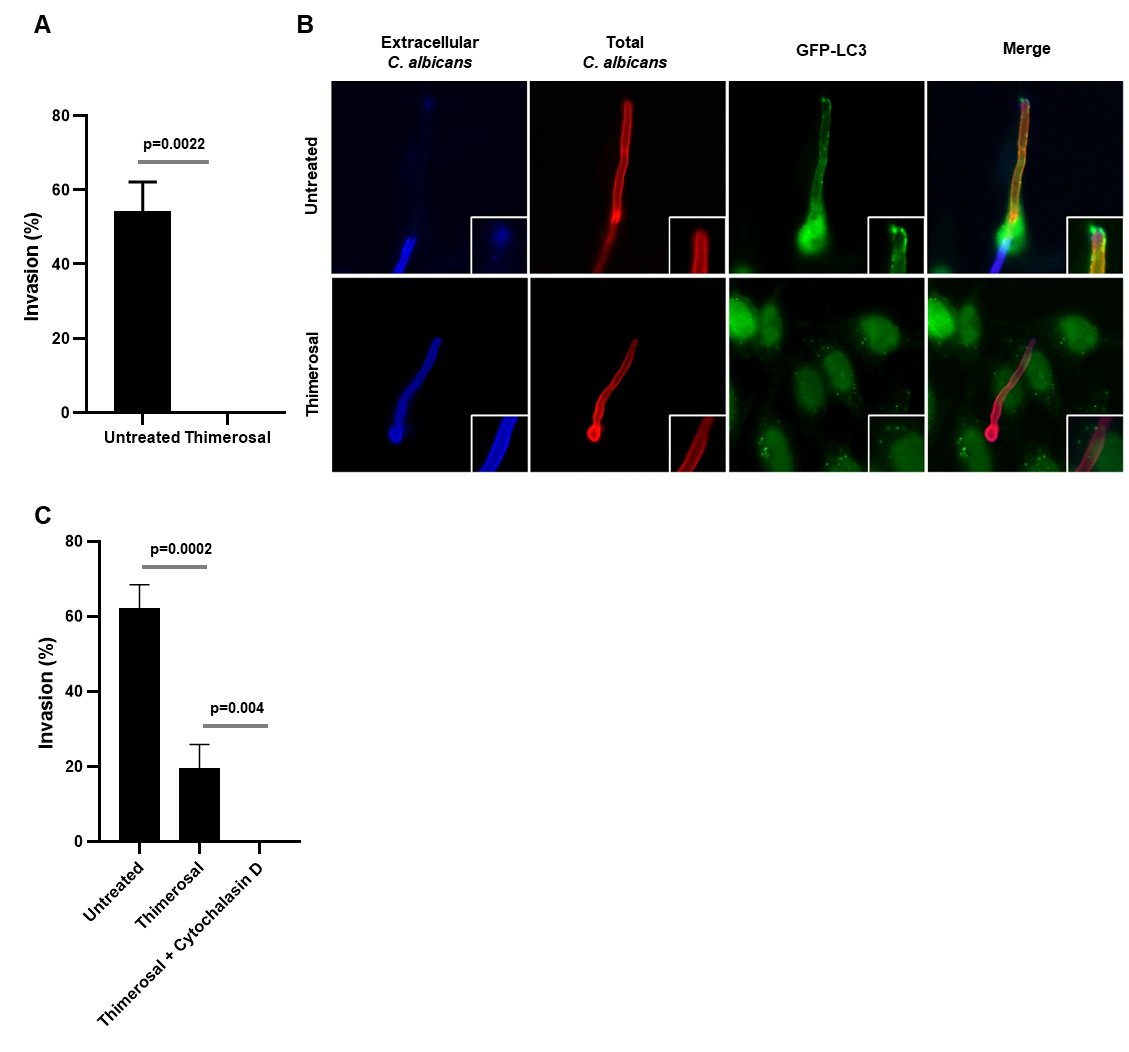


***Supplementary Figure 5:***

(**A**) Percentage of invasion of untreated or thimerosal-pretreated *C. albicans* hyphae in GFP-LC3 HeLa cells at 4 h post-infection. Each value is the mean of six independent experiments ± SEM. (**B**) Representative images of untreated or thimerosal-pretreated *C. albicans* hyphae in HeLa cells at 4 h post-infection. Samples were processed for extracellular *C. albicans* (blue), total *C. albicans* (red) and GFP-LC3 (green) staining. (**C**) Percentage of invasion at 4 h post-infection of untreated or thimerosal-pretreated *C. albicans* hyphae in HCT116 cells, pretreated or not with cytochalasin D (500 nM). Each value is the mean of four independent experiments ± SEM.


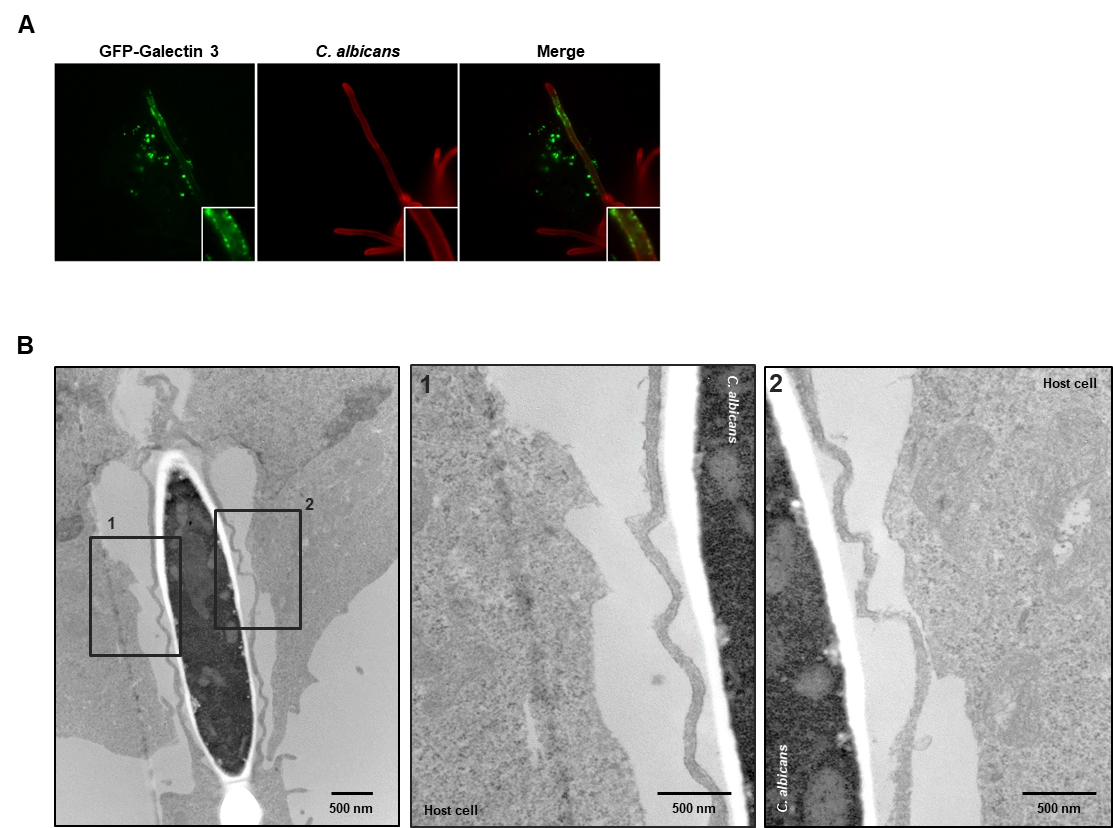


***Supplementary Figure 6:***

(**A**) Representative images of HeLa cells transfected with GFP-Galectin 3 and infected for 4 h with *C. albicans*. Samples were processed for GFP-Galectin 3 (green) and *C. albicans* (red) stainings. (**B**) Ultrastructural analysis by transmission electron microscopy of HCT116 cells infected with *C. albicans* for 4 h.


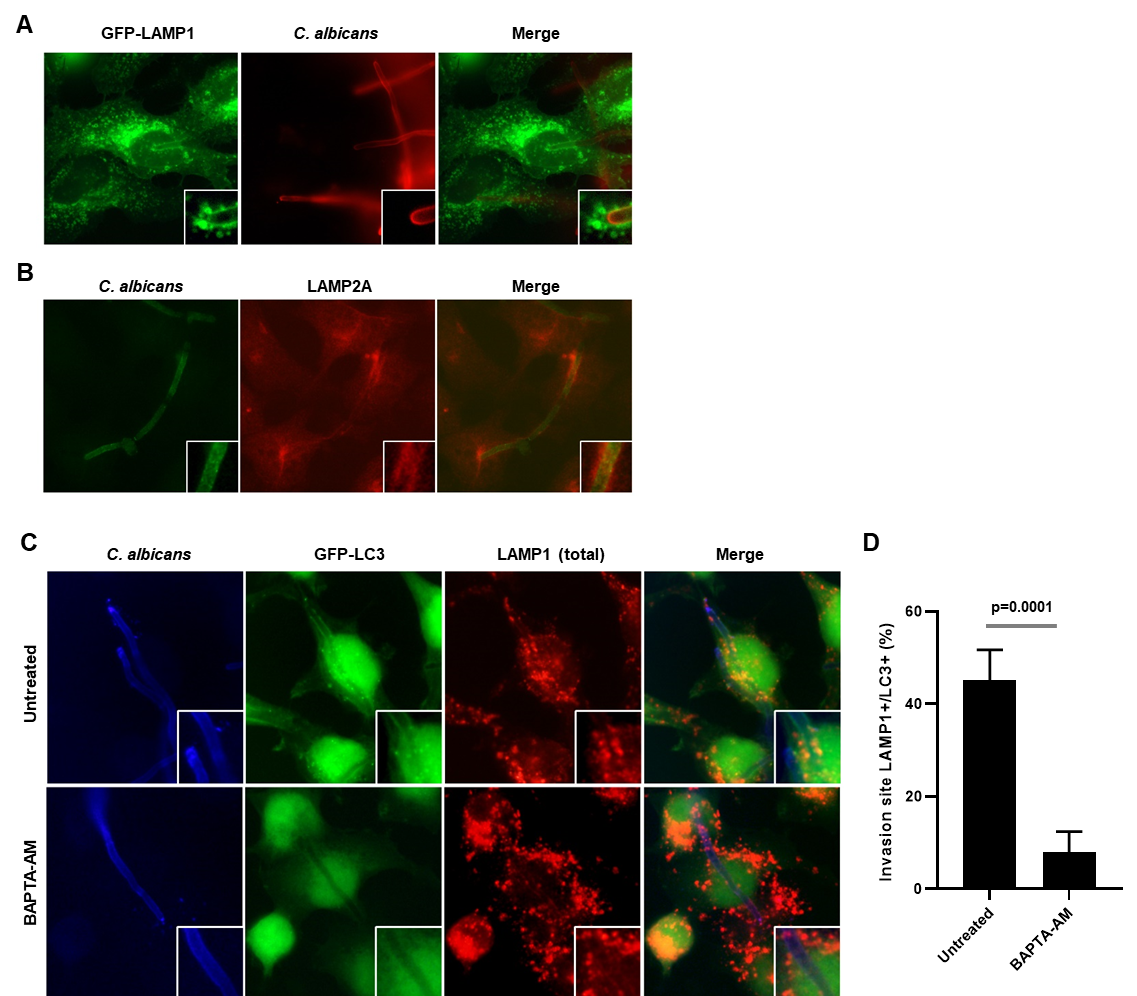


***Supplementary Figure 7:***

(**A**) Representative images of HeLa cells transfected with GFP-LAMP1 and infected for 4 h with *C. albicans*. Samples were processed for GFP-LAMP1 (green) and *C. albicans* (red) stainings. (**B**) Representative images of HeLa cells infected for 4 h with *C. albicans*. Samples were processed for *C. albicans* (green) and LAMP2A (red) stainings. (**C**) Representative images of GFP-LC3 HeLa cells, treated or not with the calcium-chelating agent BAPTA-AM (10 μM) and infected for 4 h with *C. albicans*. Samples were processed for *C. albicans* (blue), LAMP1 (red) and GFP-LC3 (green) staining. (**D**) Quantification of the percentage of *C. albicans* invasion sites double positive for GFP-LC3 and LAMP1 in untreated or BAPTA-AM-treated cells. Data are mean +/- SEM of three independent experiments.
