# Supplementary figures and images for: Membrane protective role of autophagic machinery during infection of epithelial cells by Candida albicans
Source: Gut Microbes. 2022 Jan 27;14(1):2004798. doi: 10.1080/19490976.2021.2004798 (PMC8803057; doi:10.1080/19490976.2021.2004798)

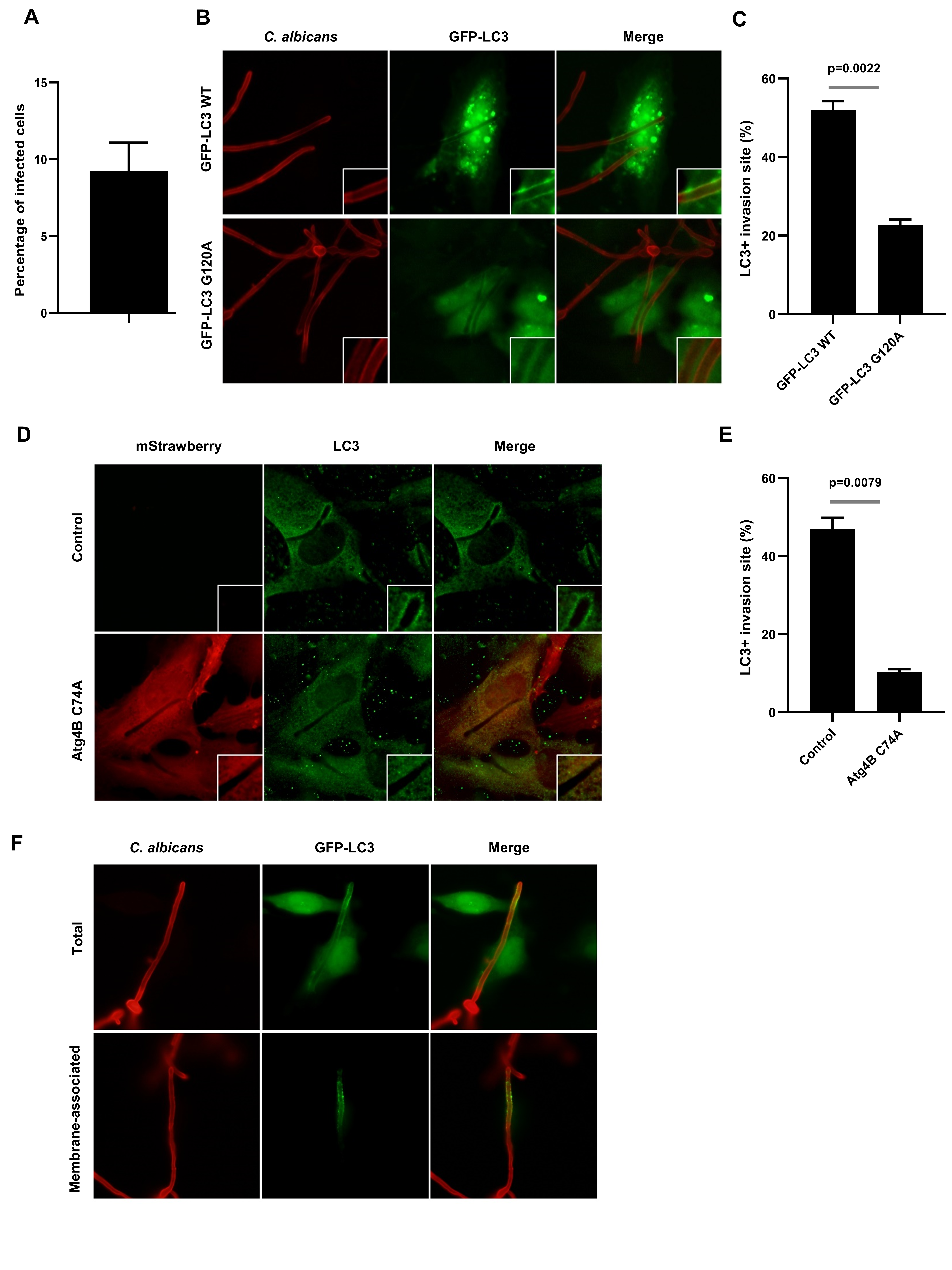

Supplement: Supplemental Material [file KGMI_A_2004798_SM7022.zip › Supplementary information/Lapaquette_et_al_SupFigure_1.TIF]

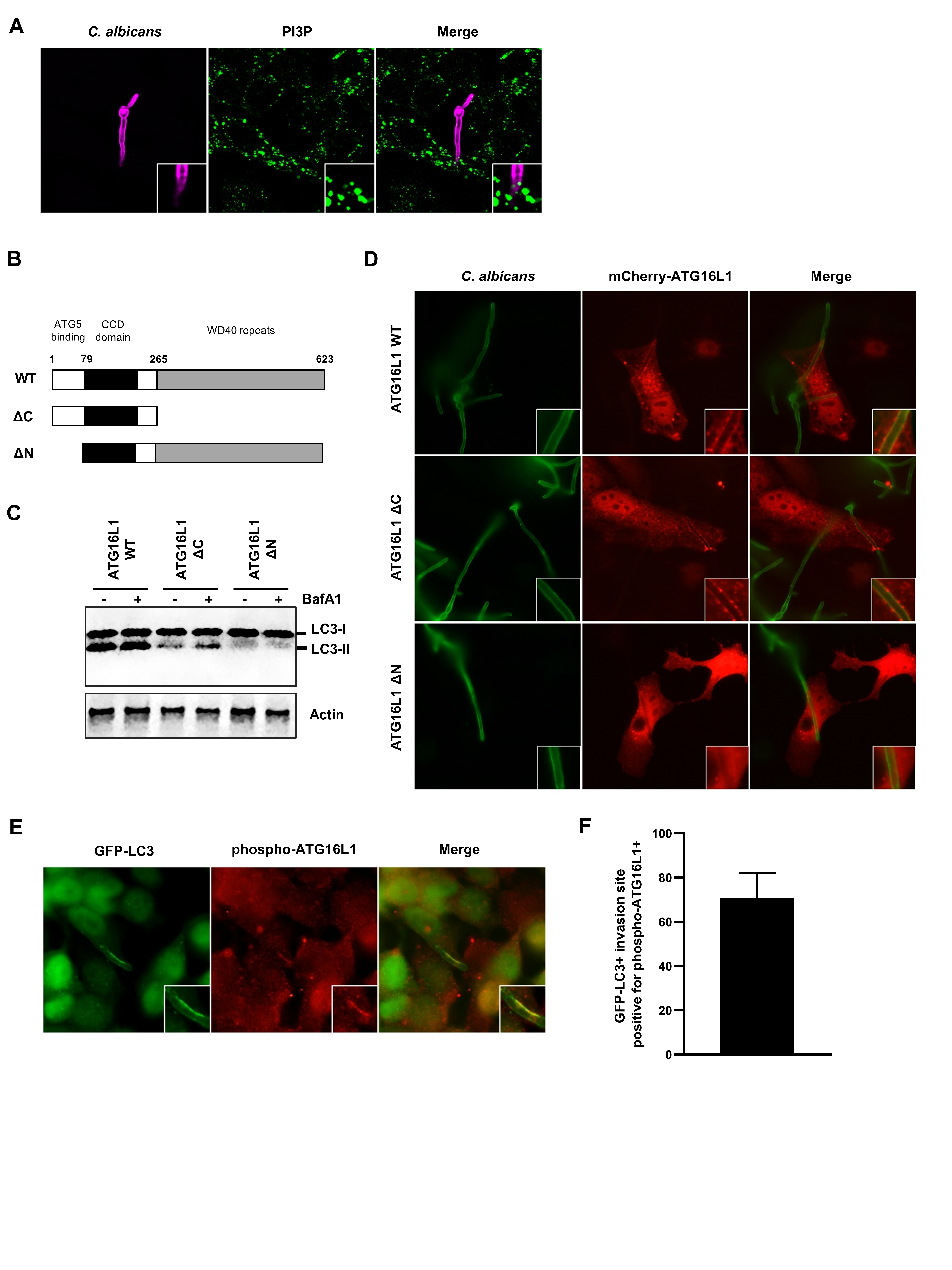

Supplement: Supplemental Material [file KGMI_A_2004798_SM7022.zip › Supplementary information/Lapaquette_et_al_SupFigure_2.TIF]

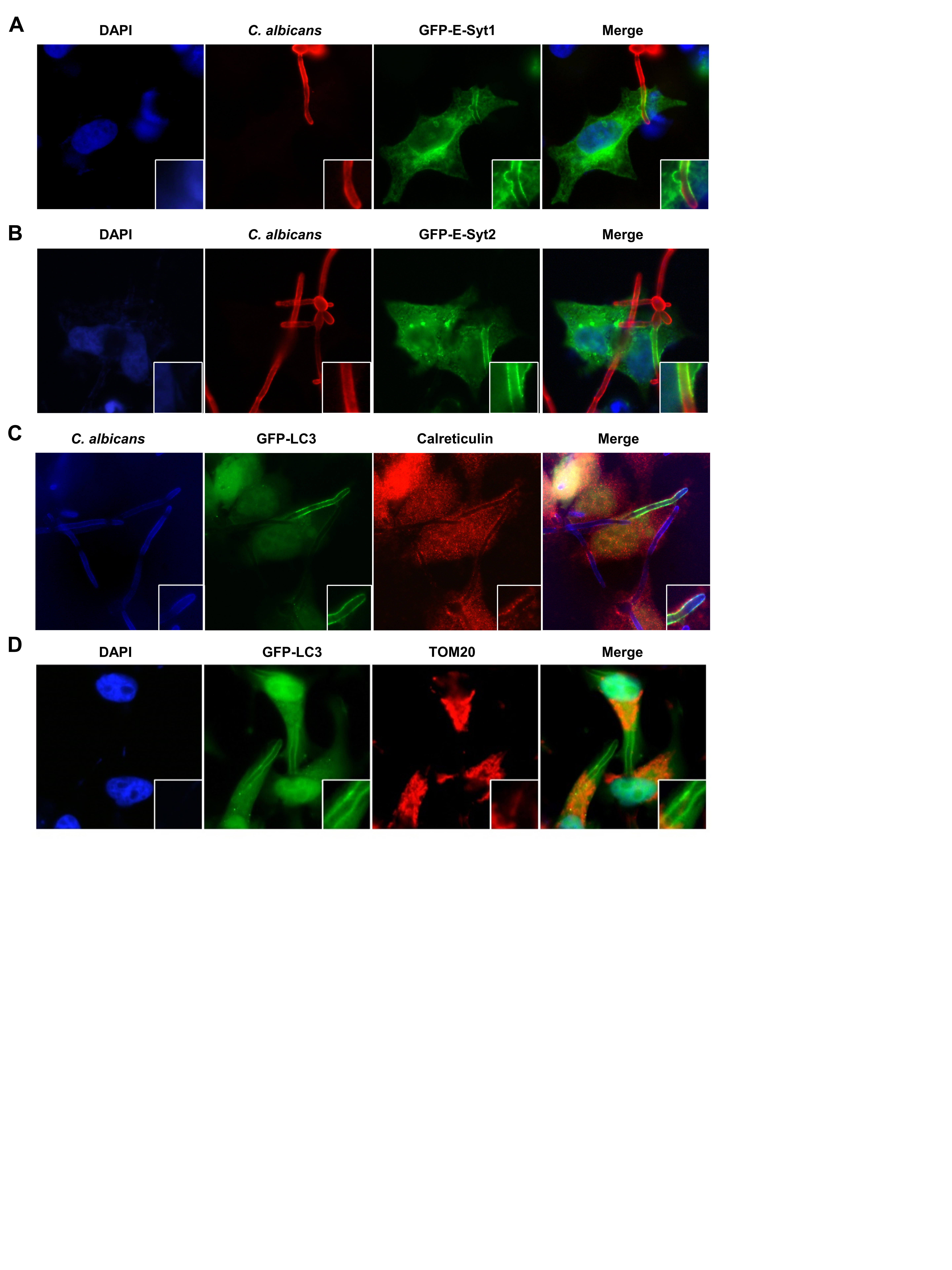

Supplement: Supplemental Material [file KGMI_A_2004798_SM7022.zip › Supplementary information/Lapaquette_et_al_SupFigure_3.TIF]

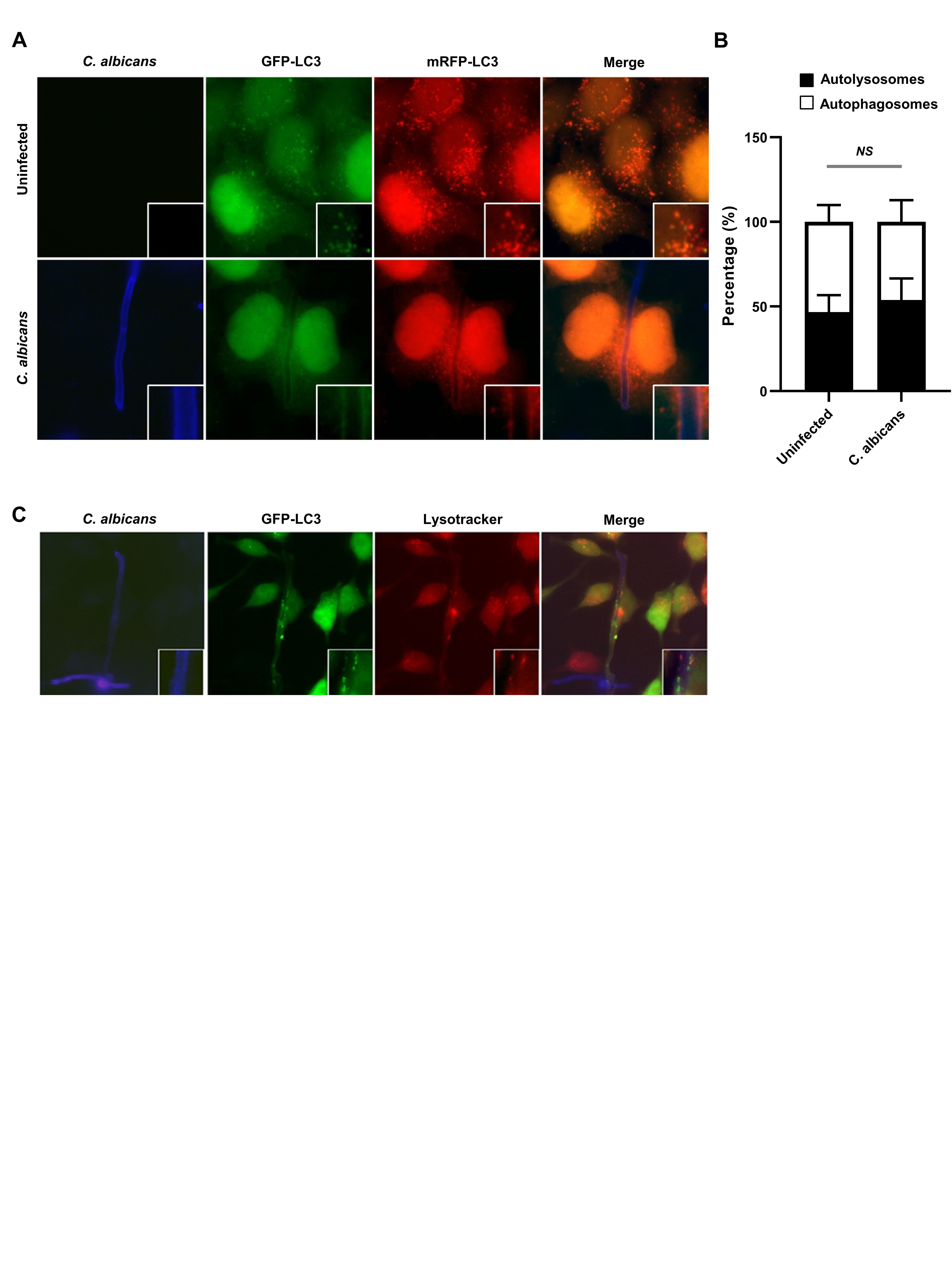

Supplement: Supplemental Material [file KGMI_A_2004798_SM7022.zip › Supplementary information/Lapaquette_et_al_SupFigure_4.TIF]

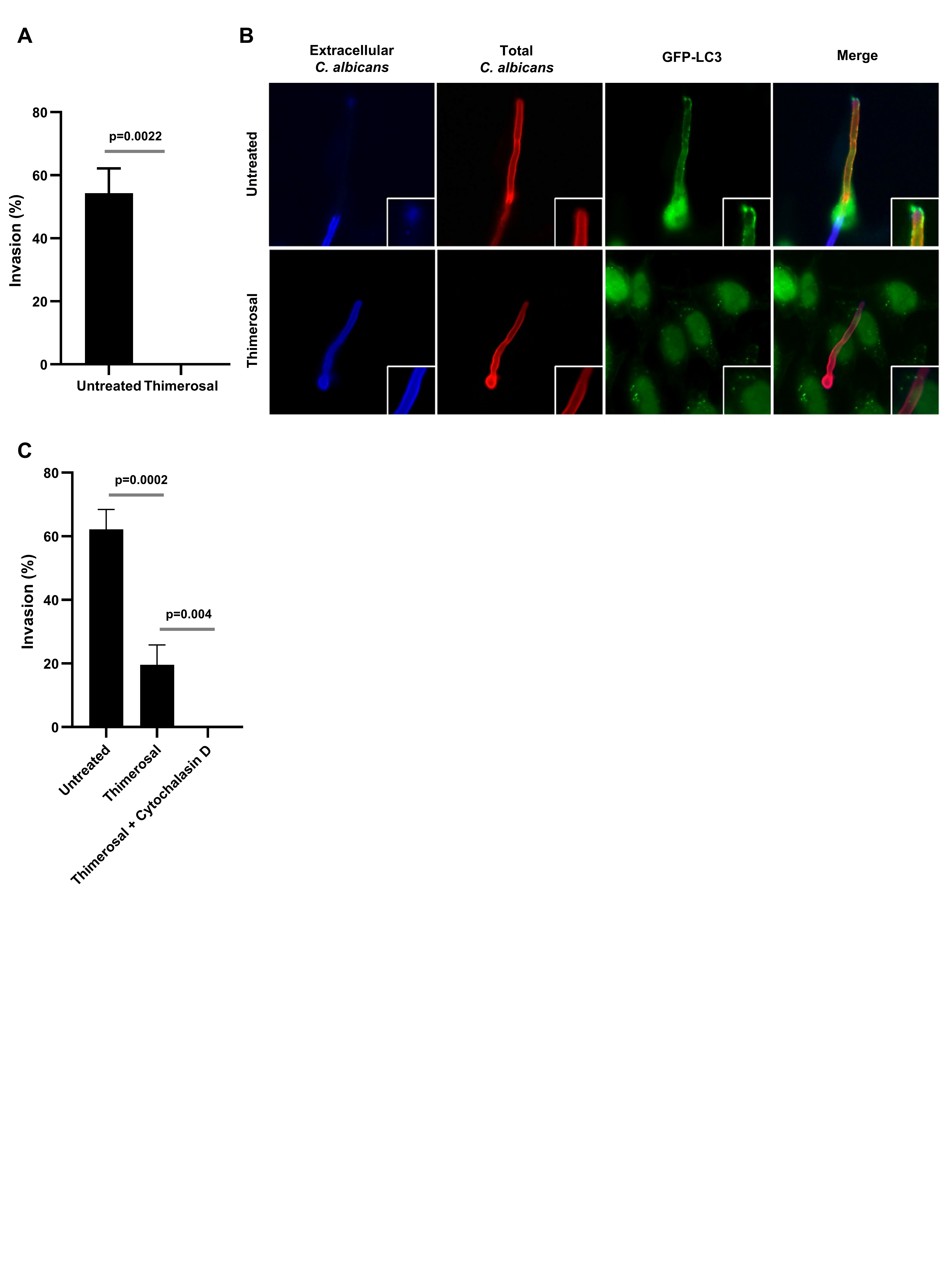

Supplement: Supplemental Material [file KGMI_A_2004798_SM7022.zip › Supplementary information/Lapaquette_et_al_SupFigure_5.TIF]

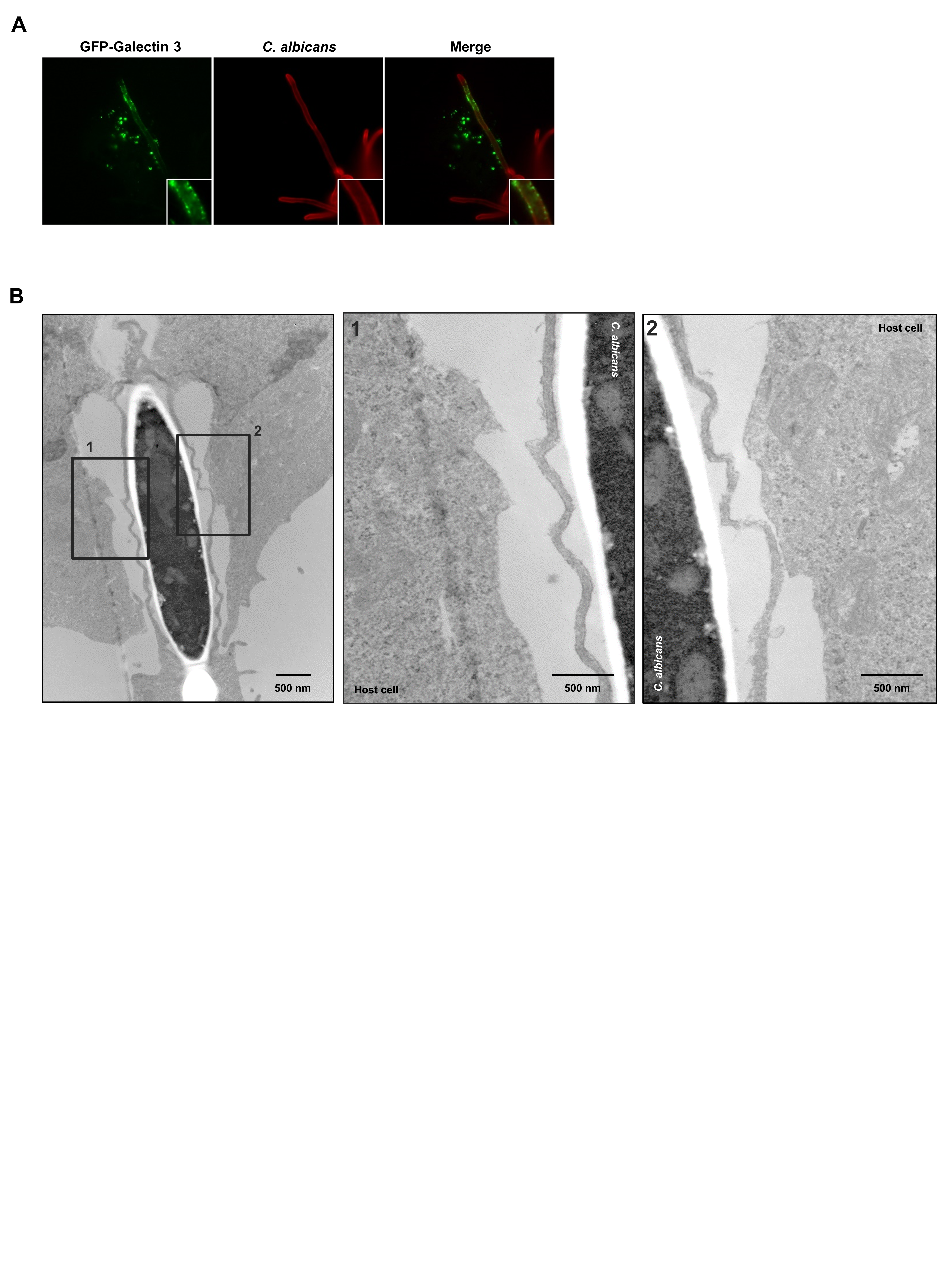

Supplement: Supplemental Material [file KGMI_A_2004798_SM7022.zip › Supplementary information/Lapaquette_et_al_SupFigure_6.TIF]

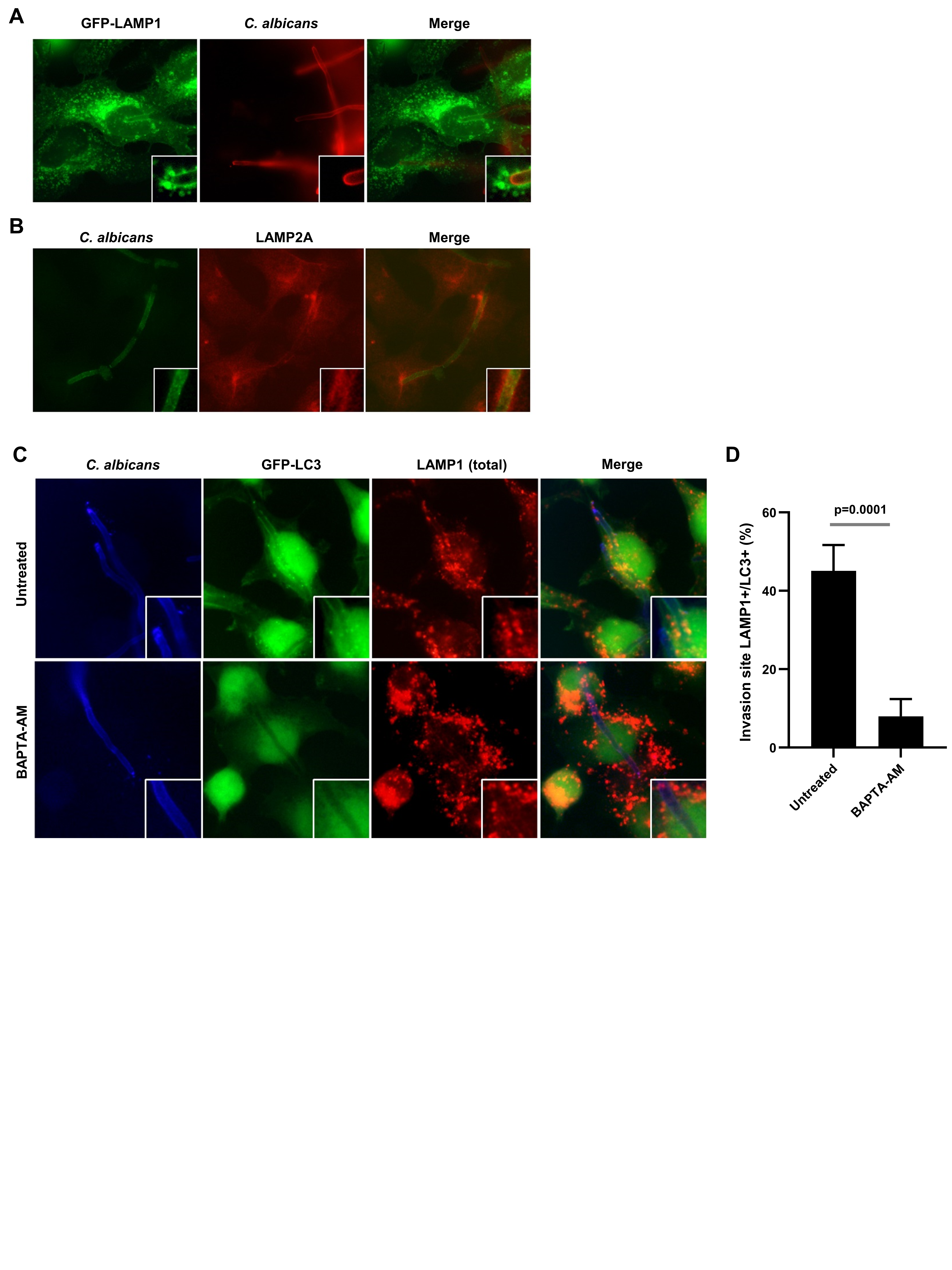

Supplement: Supplemental Material [file KGMI_A_2004798_SM7022.zip › Supplementary information/Lapaquette_et_al_SupFigure_7.TIF]
